# A YAP/TAZ-TEAD signalling module links endothelial nutrient acquisition to angiogenic growth

**DOI:** 10.1038/s42255-022-00584-y

**Published:** 2022-06-20

**Authors:** Yu Ting Ong, Jorge Andrade, Max Armbruster, Chenyue Shi, Marco Castro, Ana S. H. Costa, Toshiya Sugino, Guy Eelen, Barbara Zimmermann, Kerstin Wilhelm, Joseph Lim, Shuichi Watanabe, Stefan Guenther, Andre Schneider, Francesca Zanconato, Manuel Kaulich, Duojia Pan, Thomas Braun, Holger Gerhardt, Alejo Efeyan, Peter Carmeliet, Stefano Piccolo, Ana Rita Grosso, Michael Potente

**Affiliations:** 1 Angiogenesis & Metabolism Laboratory, Max Planck Institute for Heart and Lung Research, Bad Nauheim, Germany; 2 Berlin Institute of Health at Charité—Universitätsmedizin Berlin, Berlin, Germany; 3 Max Delbrück Center for Molecular Medicine in the Helmholtz Association, Berlin, Germany; 4 Cold Spring Harbor Laboratory, Cold Spring Harbor, NY USA; 5 Laboratory of Angiogenesis and Vascular Metabolism, Center for Cancer Biology, and Department of Oncology and Leuven Cancer Institute, VIB and KU Leuven, Leuven, Belgium; 6 Department of Cardiac Development and Remodeling, Max Planck Institute for Heart and Lung Research, Bad Nauheim, Germany; 7 Department of Molecular Medicine, University of Padua School of Medicine, Padua, Italy; 8 Institute of Biochemistry II, Goethe University, Frankfurt (Main), Germany; 9 Department of Physiology, Howard Hughes Medical Institute, University of Texas Southwestern Medical Center, Dallas, TX USA; 10 Integrative Vascular Biology Laboratory, Max Delbrück Center for Molecular Medicine in the Helmholtz Association, Berlin, Germany; 11 DZHK (German Center for Cardiovascular Research), partner site Berlin, Berlin, Germany; 12 Vascular Patterning Laboratory, Center for Cancer Biology, VIB and KU Leuven, Leuven, Belgium; 13 Metabolism and Cell Signaling Laboratory, Spanish National Cancer Research Centre, Madrid, Spain; 14 Center for Biotechnology, Khalifa University of Science and Technology, Abu Dhabi, United Arab Emirates; 15 Laboratory of Angiogenesis and Vascular Heterogeneity, Department of Biomedicine, Aarhus, Denmark; 16 IFOM-ETS, the AIRC Institute of Molecular Oncology, Milan, Italy; 17 UCIBIO – Applied Molecular Biosciences Unit, Department of Life Sciences, NOVA School of Science and Technology, Universidade NOVA de Lisboa, Caparica, Portugal; 18 Present Address: Department of Environmental Medicine and Public Health, Icahn School of Medicine at Mount Sinai, New York, NY USA

**Keywords:** Metabolism, Angiogenesis, HIPPO signalling, TOR signalling

## Abstract

Angiogenesis, the process by which endothelial cells (ECs) form new blood vessels from existing ones, is intimately linked to the tissue’s metabolic milieu and often occurs at nutrient-deficient sites. However, ECs rely on sufficient metabolic resources to support growth and proliferation. How endothelial nutrient acquisition and usage are regulated is unknown. Here we show that these processes are instructed by Yes-associated protein 1 (YAP)/WW domain-containing transcription regulator 1 (WWTR1/TAZ)-transcriptional enhanced associate domain (TEAD): a transcriptional module whose function is highly responsive to changes in the tissue environment. ECs lacking YAP/TAZ or their transcriptional partners, TEAD1, 2 and 4 fail to divide, resulting in stunted vascular growth in mice. Conversely, activation of TAZ, the more abundant paralogue in ECs, boosts proliferation, leading to vascular hyperplasia. We find that YAP/TAZ promote angiogenesis by fuelling nutrient-dependent mTORC1 signalling. By orchestrating the transcription of a repertoire of cell-surface transporters, including the large neutral amino acid transporter SLC7A5, YAP/TAZ-TEAD stimulate the import of amino acids and other essential nutrients, thereby enabling mTORC1 activation. Dissociating mTORC1 from these nutrient inputs—elicited by the loss of Rag GTPases—inhibits mTORC1 activity and prevents YAP/TAZ-dependent vascular growth. Together, these findings define a pivotal role for YAP/TAZ-TEAD in controlling endothelial mTORC1 and illustrate the essentiality of coordinated nutrient fluxes in the vasculature.

## Abstract (web summary)

Ong et al. report how YAP/TAZ and mTORC1 signalling intersect in endothelial cells to regulate nutrient acquisition and vascular growth.

## Main

Blood vessels form extensive tubular networks of arteries, capillaries and veins that nurture all body tissues. Endothelial cells (ECs) line the inner surface of these networks, where they are surrounded by diverse nutrients such as amino acids, glucose and lipids. In the resting state, ECs take up sufficient amounts of these nutrients to enable transport to perivascular tissues and to sustain their basal homeostatic needs^1,2^. However, when activated by growth factors to form new vessel branches, ECs must increase nutrient uptake and consumption to meet the metabolic demands of the angiogenic response^3,4^. In addition to adenosine triphosphate (ATP), growth factor-activated ECs need carbon, nitrogen and reducing agents to support the biosynthesis of macromolecules (for example, nucleic acids, proteins, lipids) necessary for vascular expansion^3,4^. Controlling nutrient acquisition and usage is, therefore, central to the function of the endothelium; yet, the mechanisms that regulate these processes are poorly understood.

To gain insights into this regulation, we analysed signalling by the related transcriptional cofactors YAP1 (hereafter referred to as YAP) and WWTR1 (hereafter referred to as TAZ). YAP and TAZ are effectors of the Hippo pathway and essential regulators of vascular development^5–13^, whose activity is highly sensitive to changes in the micro-environment^14–19^. By sensing and responding to mechanical, metabolic and soluble signals, these proteins coordinate tissue growth responses^14–19^.

**Fig. 1:**
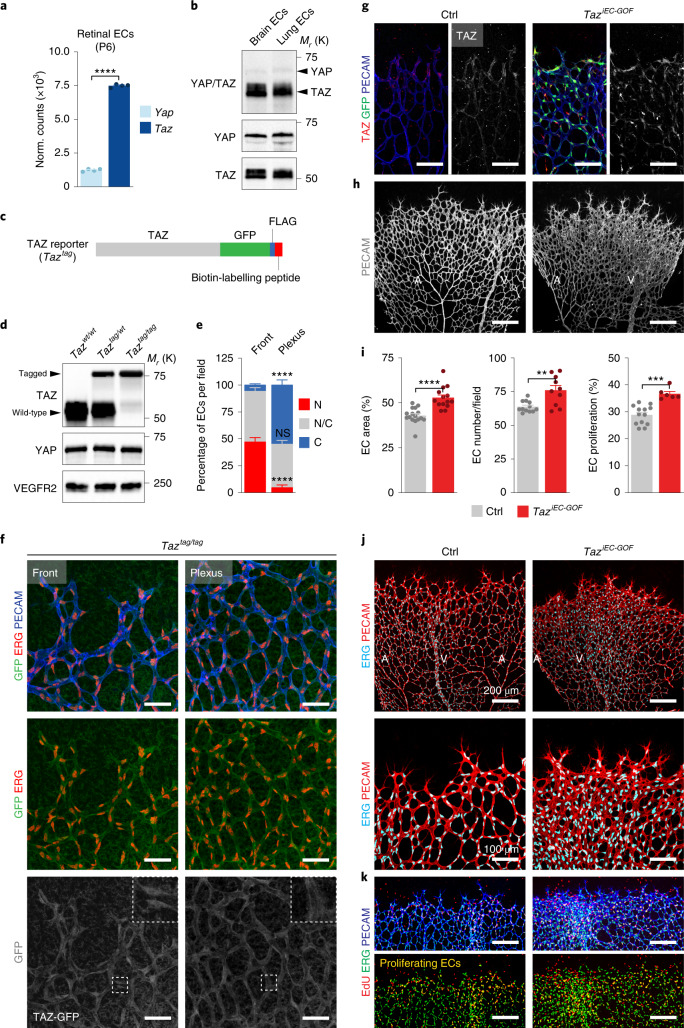
Nuclear TAZ signalling drives vascular growth. **a**, *Yap*/*Taz* transcript levels in ECs sorted from postnatal day (P) 6 mouse retinas as determined by RNA-seq (*n* = 4 independent samples). **b**, YAP/TAZ immunoblot analysis of ECs isolated from murine brains and lungs. **c**, Schematic of the *Taz*^*tag*^ reporter containing GFP, FLAG and a biotin-labelling peptide. **d**, Expression of the reporter-tagged TAZ protein in whole lung lysates of wild-type, heterozygous and homozygous *Taz*^*tag*^ mice. VEGFR2, endothelial marker. **e**, Quantification of TAZ subcellular localization in ECs of *Taz*^*tag/tag*^ P6 retinas. N, preferentially nuclear; NC, nucleo-cytoplasmic; C, preferentially cytoplasmic (*n* = 10 independent samples). **f**, Images of GFP, ERG and PECAM-labelled P6 retinas derived from *Taz*^*tag/tag*^ mice. The grey images (lower panels) show the isolated GFP signal. The small (white) boxed area is shown at higher magnification in the upper right corner. Scale bars, 50 μm. **g**, Immunolabelling for TAZ, GFP and PECAM in P6 retinas of *Taz*^*iEC-GOF*^ (*Pdgfb-CreERT2;Rosa26-Taz*^*S89A fl/fl*^) and control (Ctrl; *Rosa26-Taz*^*S89A fl/fl*^) mice. The grey images (right panels) show the isolated TAZ signal. Scale bar, 100 μm. **h**, Confocal images of PECAM-labelled P6 mouse retinas of Ctrl and *Taz*^*iEC-GOF*^ mice. A, artery; V, vein. Scale bar, 200 μm. **i**, Quantification of vascular parameters in Ctrl and *Taz*^*iEC-GOF*^ mutants as indicated (EC area, *n* = 16 (Ctrl) and 14 (*Taz*^*iEC-GOF*^) independent samples; EC number/field, *n* = 12 (Ctrl) and 10 (*Taz*^*iEC-GOF*^) independent samples; EC proliferation, *n* = 13 (Ctrl) and 6 (*Taz*^*iEC-GOF*^) independent samples). **j**, ERG and PECAM- labelled retinas at P6 showing a hyperplastic vasculature in *Taz*^*iEC-GOF*^ mice. **k**, Immunofluorescence images of the angiogenic front in P6 retinas of Ctrl and *Taz*^*iEC-GOF*^ mice labelled for EdU, ERG and PECAM-. Scale bar, 200 μm. Western blot data in **b** and **d** are from the respective experiment, processed in parallel and are representative of three independent experiments. For **a**,**d**,**e** and **i**, data represent mean ± s.e.m.; two-tailed unpaired *t*-test was used. **P < 0.01; ***P < 0.001; ****P < 0.0001; NS, not significant. The numerical data, unprocessed western blots and *P* values are provided as source data. Source data

**Extended Data Fig. 1:**
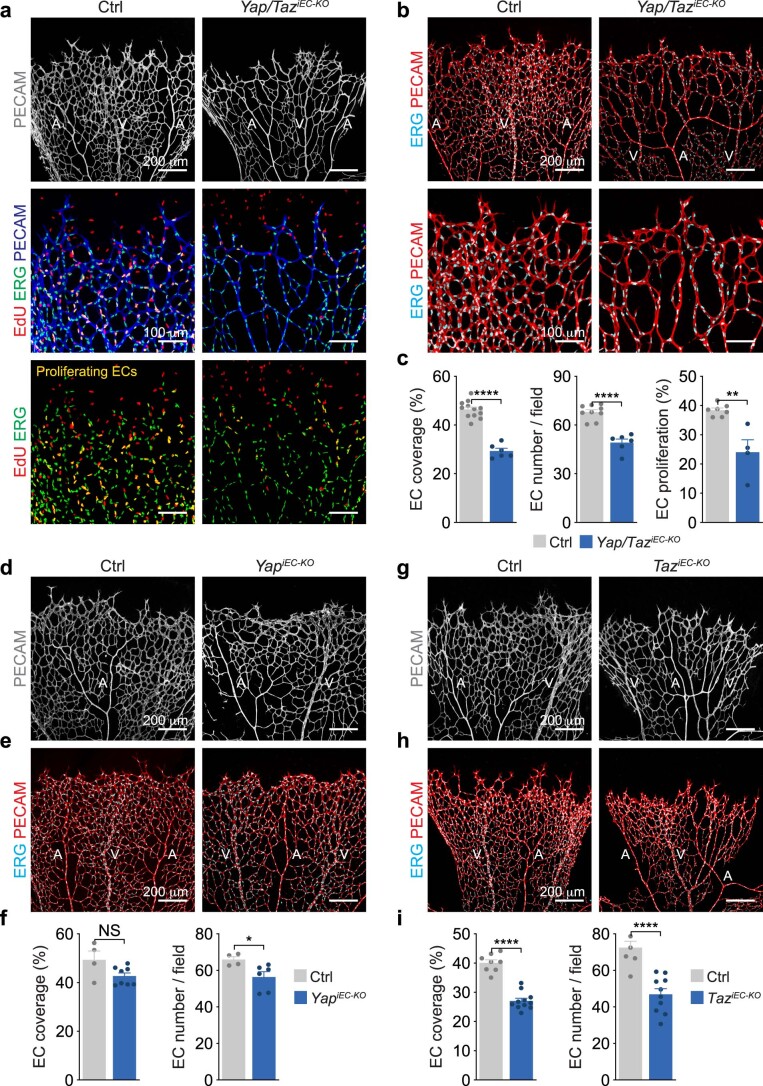
Inactivation of endothelial TAZ mimics several of the YAP/TAZ mutant phenotypes. **a**, EdU, ERG and PECAM immunofluorescence labelling of P6 mouse retinas depicting reduced vascular density and impaired endothelial proliferation in *Yap/Taz*^*iEC-KO*^ (*Pdgfb-CreERT2;Yap*^*fl/fl*^*/Taz*^*fl/fl*^) mice following injection of 4OHT from P1-P4. 4OHT-treated *Yap*^*fl/fl*^*/Taz*^*fl/fl*^ mice served as control (Ctrl). A, artery; V, vein. The images of the isolated PECAM signal are shown in grey at lower magnification in the upper part of the panel. **b**, Confocal images of ERG and PECAM labelled P6 retinas of Ctrl and *Yap/Taz*^*iEC-KO*^ mice. **c**, Quantification of vascular parameters in Ctrl and *Yap/Taz*^*iEC-KO*^ mice at P6 as indicated (EC coverage: n = 12 (Ctrl) and 6 (*Yap/Taz*^*iEC-KO*^) independent samples; EC number/field: n = 9 (Ctrl) and 6 (*Yap/Taz*^*iEC-KO*^) independent samples; EC proliferation: n = 7 (Ctrl) and 4 (*Yap/Taz*^*iEC-KO*^) independent samples). **d**-**f**, Confocal images (**d,e**) and quantifications (**f)** of PECAM (**d**) and ERG and PECAM (**e**) labelled Ctrl (*Yap*^*fl/fl*^) and *Yap*^*iEC-KO*^ (*Pdgfb-CreERT2;Yap*^*fl/fl*^) retinas at P6 (EC coverage: n = 4 (Ctrl) and 8 (*Yap*^*iEC-KO*^) independent samples; EC number/field: n = 4 (Ctrl) and 6 (*Yap*^*iEC-KO*^) independent samples). **g**-**i**, Confocal images (**g**,**h**) and quantifications (**i**) of PECAM (**g**) and ERG and PECAM (**h**) labelled Ctrl (*Taz*^*fl/fl*^) and *Taz*^*iEC-KO*^ (*Pdgfb-CreERT2;Taz*^*fl/fl*^) retinas at P6 (EC coverage: n = 8 (Ctrl) and 11 (*Taz*^*iEC-KO*^) independent samples; EC number/field: n = 5 (Ctrl) and 10 (*Taz*^*iEC-KO*^) independent samples). For **c**, **f** and **i**, data represent mean ± s.e.m.; two-tailed unpaired t-test. *P < 0.05; **P < 0.01; ****P < 0.0001; NS, not significant. The numerical data and *P* values are provided as source data. Source data

**Extended Data Fig. 2:**
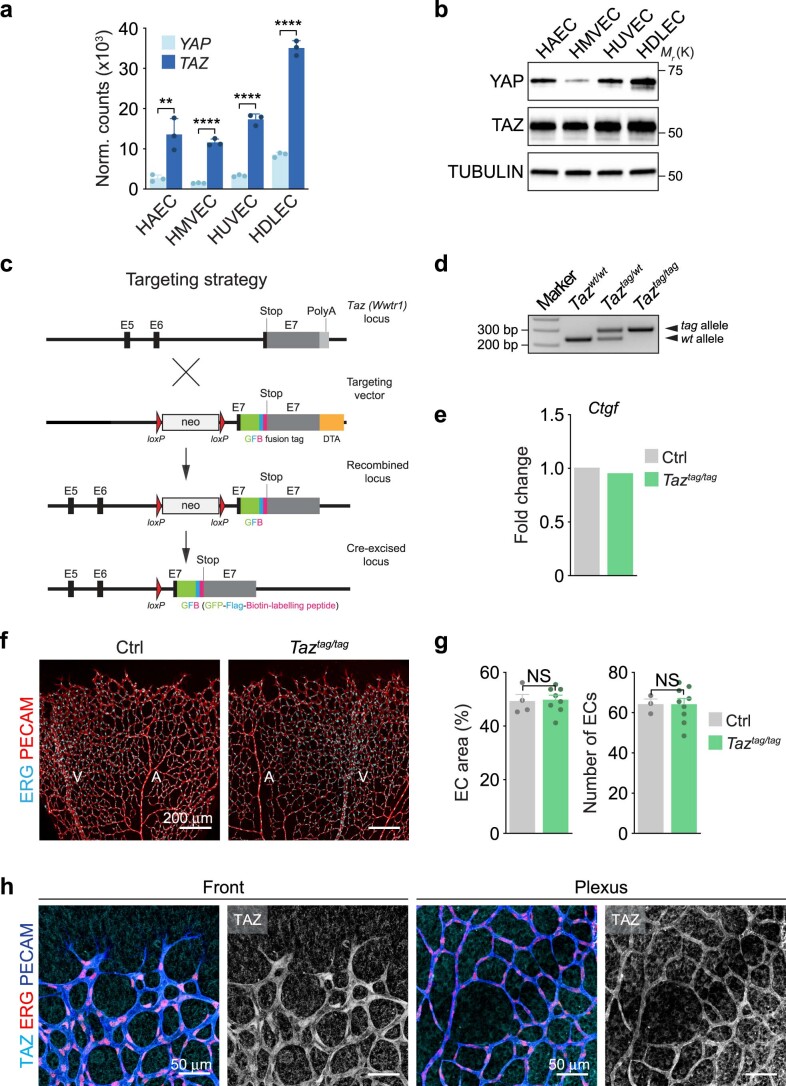
Expression and subcellular localization of TAZ in angiogenic ECs. **a**, RNA-seq analysis of *YAP* and *TAZ* transcript levels in different human EC cultures (n = 3 independent samples). HAECs, human aortic ECs; HMVECs, human microvascular ECs; HUVECs, human umbilical vein ECs; HDLEC, human dermal lymphatic ECs. **b**, Immunoblot analysis of YAP and TAZ protein expression in different endothelial subtypes. **c**, Targeting strategy to generate a *Taz* (*Wwtr1*) knock-in allele encoding for a GFP, FLAG, and biotin-labelling peptide tagged TAZ protein. A schematic representation of the wild-type *Taz* (*Wwtr1*) locus, the targeting vector, the recombined as well as the excised locus is shown. E5^−^7, exons 5 to 7. Triangles denote *loxP* sites. Neo, neomycin positive selection cassette. GFB, GFP-FLAG-biotin labelling peptide fusion tag. DTA, diphteria toxin negative selection marker. **d**, PCR analysis of genomic DNA obtained from wild-type, heterozygote and homozygote *Taz*^*tag*^ mice. Lane 1, DNA marker. **e**, RT-qPCR analysis of the canonical YAP/TAZ target gene *Ctgf* in retinal ECs derived from wild-type (Ctrl) and homozygous *Taz*^*tag/tag*^ mice, showing no expression difference between the two genotypes (n = 2 (Ctrl) and 3 (*Taz*^*tag/tag*^) independent samples). **f**, **g**, ERG- and PECAM- immunofluorescence staining (**f**) and quantification of vascular parameters (**g**) of P6 Ctrl and *Taz*^*tag/tag*^ retinas, revealing no gross difference in vascular morphogenesis between the two genotypes (EC area: n = 4 (Ctrl) and 8 (*Taz*^*tag/tag*^) independent samples; Number of ECs: n = 3 (Ctrl) and 9 (*Taz*^*tag/tag*^) independent samples). **h**, Images of the vascular front and plexus in TAZ, ERG and PECAM labelled P6 mouse retinas derived from C57BL/6 wild-type mice. The images in grey (right panels) show the isolated TAZ signal. Western blot data in **b** are from the respective experiment, processed in parallel, and are representative of at least three independent experiments. For **a**, **e** and **g**, data represent mean ± s.e.m.; two-tailed unpaired t-test. **P < 0.01; ****P < 0.0001; NS, not significant. The numerical data, unprocessed western blots and *P* values are provided as source data. Source data

We bred mice carrying floxed alleles of both cofactors (*Yap*^*fl/fl*^;*Taz*^*fl/fl*^)^20,21^ and expressed the tamoxifen-inducible recombinase *CreERT2* driven by the endothelial-restricted platelet derived growth factor subunit B (*Pdgfb*) promoter (*Yap;Taz*^*iEC-KO*^). Analysis of the developing retinal vasculature in these mutants revealed severe angiogenic defects after 4-hydroxy-tamoxifen (4OHT) administration, as reported^5–9^ (Extended Data Fig. 1a–c). Compared to controls, *Yap;Taz*^*iEC-KO*^ mice had fewer and less proliferative ECs, giving rise to a sparse and mis-patterned vascular network with poor connectivity (Extended Data Fig. 1a–c). Deletion of *Taz* alone mimicked most of these vascular phenotypes, while deletion of *Yap* had little effect (Extended Data Fig. 1d–i). Compared to YAP, TAZ was also the more abundant transcript in various endothelial subtypes (Fig. 1a,b and Extended Data Fig. 2a,b), suggesting a critical role of TAZ for YAP/TAZ responses in the endothelium.

To study the role of TAZ in ECs further, we engineered a *Taz* reporter mouse, in which a green fluorescent protein (GFP) as well as FLAG and a biotin-labelling peptide were fused to the C terminus of the endogenous TAZ protein (*Taz*^*tag*^) (Fig. 1c and Extended Data Fig. 2c,d). Mice homozygous for this knock-in mutation (*Taz*^*tag/tag*^) expressed the reporter-tagged fusion instead of the wild-type protein, had normal vascular patterning and transcript levels of prototypic YAP/TAZ target genes were unperturbed (Fig. 1d and Extended Data Fig. 2e–g), indicating that the reporter tag does not alter TAZ function. Analysis of the GFP signal in platelet EC adhesion molecule (PECAM) (marking the surface of ECs) and ETS transcription factor ERG (marking endothelial nuclei) colabelled retinas confirmed high expression of TAZ in the endothelium and revealed differences in its subcellular localization (Fig. 1e,f). At the vascular front, where ECs actively divide, migrate and rearrange, TAZ showed a preferentially nuclear pattern, whereas in the central parts, where ECs are less active, TAZ was mostly cytoplasmic (Fig. 1e,f). Similar results were obtained in wild-type retinas labelled with a TAZ antibody (Extended Data Fig. 2h), suggesting dynamic regulation of TAZ subcellular localization during angiogenic growth.

**Extended Data Fig. 3:**
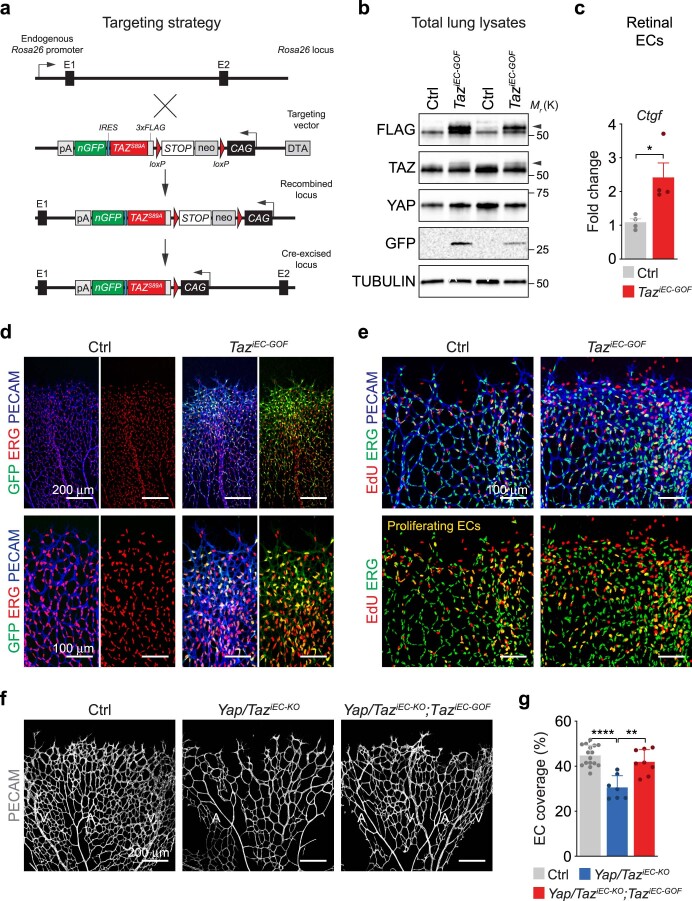
Generation and validation of conditional *Taz* gain-of-function mice. **a**, Schematic representations of the wild-type *Rosa26* locus, the targeting vector, the recombined as well as the excised allele are shown. A cassette containing the *CAG* promoter, a floxed *STOP* sequence, a cDNA encoding for *3xFLAG-tagged TAZ*^*S89A*^ and *IRES-nGFP* was inserted into the *Rosa26* locus. Triangles denote *loxP* sites. DTA, diphteria toxin negative selection marker; pA, polyA signal. **b**, Immunoblot analysis of total lung lysates from control (Ctrl, *Rosa26-Taz*^*S89A fl/fl*^) and mutant mice (*Taz*^*iEC-GOF*^, *Pdgfb-creERT2;Rosa26-Taz*^*S89A fl/fl*^). 4OHT was administered from P1 to P4 and samples analyzed at P6. Arrow heads indicate expression of the FLAG-tagged *Taz*^*S89A*^ mutant. **c**, RT-qPCR analysis of retinal ECs at P6, showing increased expression of the canonical YAP/TAZ target gene *Ctgf* in *Taz*^*iEC-GOF*^ mice when compared to Ctrl (n = 4 independent samples). **d**, Immunofluorescence staining for GFP, ERG and PECAM in P6 retinas depicting a nuclear GFP signal in PECAM positive vessels of *Taz*^*iEC-GOF*^ mutants, which is absent in Ctrl mice. **e**, Higher magnification images of P6 retinas labelled for EdU, ERG and PECAM showing increased EC proliferation in the *Taz*^*iEC-GOF*^ mice. **f**, **g**, Confocal images (**f**) and quantification of endothelial coverage (**g**) in PECAM labelled P6 retinas obtained from Ctrl (*Yap*^*fl/fl*^*;Taz*^*fl/fl*^*;Rosa26-Taz*^*S89A fl/wt*^), *Yap/Taz*^*iEC-KO*^ (*Pdgfb-creERT2;Yap*^*fl/fl*^*;Taz*^*fl/fl*^*;Rosa26-Taz*^*S89A wt/wt*^) and *Yap/Taz*^*iEC-KO*^;*Taz*^*iEC-GOF*^ (*Pdgfb-creERT2;Yap*^*fl/fl*^*;Taz*^*fl/fl*^*;Rosa26-Taz*^*S89A fl/wt*^) mice (EC coverage: n = 16 (Ctrl), 7 (*Yap/Taz*^*iEC-KO*^) and 8 (*Yap/Taz*^*iEC-KO*^;*Taz*^*iEC-GOF*^) independent samples). Western blot data in **b** are from the respective experiment, processed in parallel, and are representative of at least three independent experiments. For **c, g**, data represent mean ± s.e.m.; two-tailed unpaired t-test. *P < 0.05; **P < 0.01; ****P < 0.0001. The numerical data, unprocessed western blots and *P* values are provided as source data. Source data

To understand the functional consequences of this regulation, we generated *Rosa26* knock-in mice expressing a nuclear-localized TAZ mutant on Cre-mediated recombination (Extended Data Fig. 3a,b). This mutant has the phospho-acceptor site serine 89 replaced by alanine (*Rosa26-Taz*^*S89A fl/fl*^), rendering TAZ insensitive to cytoplasmic sequestration induced by the Hippo kinases LATS1/2 (refs. ^14,18^). *Pdgfb-CreERT2*-mediated expression of *Taz*^*S89A*^ (*Taz*^*iEC-GOF*^) in the retinal endothelium resulted in increased levels of nuclear TAZ as well as increased expression of YAP/TAZ target genes (Fig. 1g and Extended Data Fig. 3c,d), which correlated with a dense and hyperplastic vascular network (Fig. 1h–j). Endothelial 5-ethynyl-2′-deoxyuridine (EdU) incorporation was also increased in these mice (Fig. 1i,k and Extended Data Fig. 3e), suggesting that nuclear TAZ signalling is crucial for EC proliferation. Consistent with this finding, expression of *Taz*^*S89A*^ in *Yap;Taz*^*iEC-KO*^ mutants was sufficient to restore vascular density in these mice, while some of the patterning defects remained unchanged (Extended Data Fig. 3f,g).

**Fig. 2:**
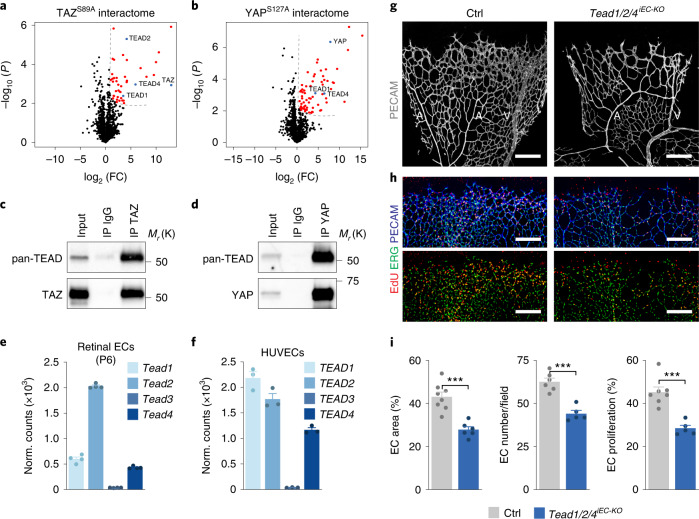
TEADs are redundant transcriptional effectors of endothelial YAP/TAZ signalling. **a**,**b**, Volcano plots of proteins interacting with FLAG-tagged TAZ^S89A^ (**a**) or YAP^S127A^ (**b**) in HUVECs (*n* = 3 independent samples). Red dots denote proteins that are significantly enriched in the TAZ^S89A^ or YAP^S127A^ interactome (log_2_ fold change (FC) ≥ 1 and false discovery rate (FDR) < 0.05). **c**,**d**, Immunoblot analysis of endothelial TAZ (**c**) and YAP (**d**) immunoprecipitates validating the interaction of endogenous YAP/TAZ with TEADs. **e**, mRNA expression profile of *Tead1–4* in murine ECs isolated from P6 mouse retinas as determined by RNA-seq analysis (*n* = 4 independent samples). **f**, Transcript abundance of *TEAD1–4* in HUVECs as assessed by RNA-seq (*n* = 3 independent samples). **g**, PECAM-immunofluorescence labelling of P6 retinas illustrating a sparse vascular network in mice lacking expression of *Tead1*, *Tead2* and *Tead4* in ECs (*Pdgfb-CreERT2;Tead1*^*fl/fl*^*;Tead2*^*−/−*^*;Tead4*^*fl/fl*^). **h**, Reduced endothelial proliferation in *Tead1/2/4*^*iEC-KO*^ mutants as shown by EdU, ERG and PECAM colabelling of P6 retinas. Scale bars in **g**,**h**, 200 μm. **i**, Quantification of vascular parameters in Ctrl and *Tead1/2/4*^*iEC-KO*^ mice (EC area, *n* = 8 (Ctrl) and 6 (*Tead1/2/4*^*iEC-KO*^) independent samples; EC number/field, *n* = 6 (Ctrl) and 5 (*Tead1/2/4*^*iEC-KO*^) independent samples; EC proliferation, *n* = 7 (Ctrl) and 5 (*Tead1/2/4*^*iEC-KO*^) independent samples). Western blot data in **c** and **d** are from the respective experiment, processed in parallel and are representative of at least three independent experiments. For **e**, **f** and **i**, data represent mean ± s.e.m.; two-tailed unpaired *t*-test. ***P < 0.001. The numerical data, unprocessed western blots and *P* values are provided as source data. Source data

In the nucleus, YAP and TAZ can interact with various transcription factors to control the expression of their target genes^14,15,18^. To identify such transcriptional regulators in ECs, we first examined proteins coimmunoprecipitating with TAZ. To this end, we transduced human umbilical vein ECs (HUVECs) with FLAG-tagged TAZ^S89A^ (AdTAZ^S89A^) or GFP as a control (AdCtrl) and performed immunoprecipitations with a FLAG antibody. Mass spectrometry analysis revealed that the TEAD proteins were the most significantly enriched transcription factors in the TAZ^S89A^ interactome under these conditions (log_2_ FC > 1, FDR < 0.05) (Fig. 2a and Supplementary Table 1). Similar results were obtained when we analysed proteins that interact with a nuclear form of YAP (YAP^S127A^) (Fig. 2b and Supplementary Table 1) or when the interactions were assessed between endogenous proteins (Fig. 2c,d), suggesting that TEADs are a central transcriptional platform through which endothelial YAP/TAZ signal.

**Extended Data Fig. 4:**
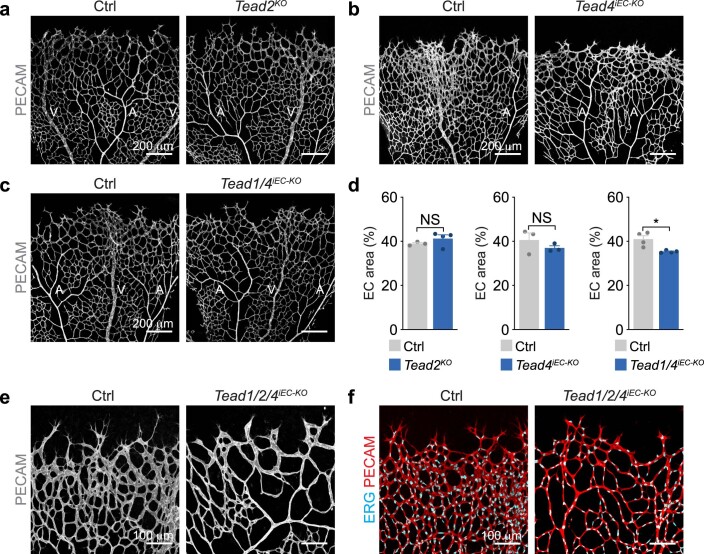
Vascular phenotypes of *Tead* mutant mice in the postnatal mouse retina. **a**-**c**, PECAM immunofluorescence labelling of P6 (**a**) *Tead2*^*KO*^ (*Tead2*^*-/-*^), (**b**) *Tead4*^*iEC-KO*^ (*Pdgfb-CreERT2;Tead4*^*fl/fl*^) and (**c**) *Tead1/4*^*iEC-KO*^ (*Pdgfb-CreERT2;Tead1*^*fl/fl*^*;Tead4*^*fl/fl*^) retinas, showing only mild vascular phenotypes when compared to controls (*Tead2*^*+/+*^, *Tead4*^*fl/fl*^ and *Tead1*^*fl/fl*^*;Tead4*^*fl/fl*^, respectively). **d**, Quantification of endothelial coverage in Ctrl, *Tead2*^*KO*^, *Tead4*^*iEC-KO*^ and *Tead1/4*^*iEC-KO*^ mice (EC area: n = 3 (Ctrl) and 4 (*Tead2*^*KO*^); EC area: n = 3 (Ctrl) and 3 (*Tead4*^*iEC-KO*^); EC area: n = 4 (Ctrl) and 4 (*Tead1/4*^*iEC-KO*^) independent samples). **e**, Higher magnification images of P6 retinas from Ctrl and *Tead1/2/4*^*iEC-KO*^ mice, showing a sparse capillary network with clumped endothelial sprouts in the mutants. **f**, Confocal images of ERG and PECAM stained P6 retinas in Ctrl and *Tead1/2/4*^*iEC-KO*^ mice, revealing a hypocellular endothelial network in the endothelial *Tead* mutant retinas. For **d**, data represent mean ± s.e.m.; two-tailed unpaired t-test. *P < 0.05; NS, not significant. The numerical data and *P* values are provided as source data. Source data

To confirm this hypothesis, we conditionally eliminated *Tead1*, *Tead2* and *Tead4* in ECs of mice: the three TEAD family members that interact with YAP/TAZ (Fig. 2a,b and Supplementary Table 1) and are expressed by the endothelium (Fig. 2e,f)^22^. In accordance with a functional overlap^23^, genetic inactivation of individual TEADs only had a minor impact on retinal angiogenesis (Extended Data Fig. 4a–d). Instead, combined deletion of all three (*Pdgfb-CreERT2;Tead1*^*fl/fl*^*;Tead2*^*−/−*^*;Tead4*^*fl/fl*^) led to profound vascular changes characterized by a sparse and hypocellular network with fewer proliferating ECs (Fig. 2g–i and Extended Data Fig. 4e,f). Of note, the phenotypes in these mutants (*Tead1/2/4*^*iEC-KO*^) were similar to those of the *Yap;Taz*^*iEC-KO*^ mice (Extended Data Fig. 1a–c), validating TEADs as crucial transcriptional effectors of endothelial YAP/TAZ signalling.

**Fig. 3:**
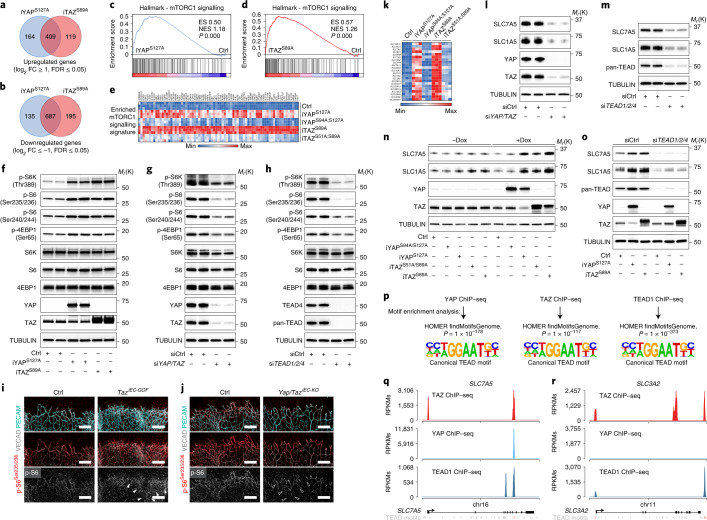
YAP/TAZ-TEAD fuel endothelial mTORC1 activity by orchestrating the transcription of nutrient transporters. **a**,**b**, Venn diagrams of up- (**a**) or downregulated (**b**) genes (log_2_ fold change (FC) ≥1 and FDR ≤ 0.05) in HUVECs transduced with inducible YAP^S127A^ (iYAP^S127A^), TAZ^S89^ (iTAZ^S89A^) or control (Ctrl) lentiviruses as assessed by RNA-seq. **c**,**d**, Gene set enrichment analysis plots depicting an enrichment of genes associated with activated mTORC1 signalling in HUVECs expressing iYAP^S127A^ (**c**) or iTAZ^S89A^ (**d**). ES, enrichment score; NES, normalized enrichment score. **e**, Heatmap of the enriched ‘mTORC1 signalling’ genes showing induction of these transcripts by iYAP^S127A^ and iTAZ^S89A^ but not by the TEAD-binding-deficient iYAP^S94A/S127A^ iTAZ^S51A/S89A^ mutants (*n* = 3 independent samples). **f**, Immunoblot analysis of S6K, S6 and 4EBP1 in Ctrl, iYAP^S127A^ and iTAZ^S89A^ transduced HUVECs, assessing phosphorylation at mTORC1-sensitive sites. **g**,**h**, Phosphorylation status of S6K, S6 and 4EBP1 in HUVECs that were transfected with siRNAs targeting *YAP*/*TAZ* (si*YAP/TAZ*) (**g**) or *TEAD1*/*TEAD2/TEAD4* (si*TEAD1/2/4*) (**h**). **i**,**j**, Immunolabelling of p-S6^Ser235/236^, VECAD and PECAM in P6 retinas of Ctrl, *Taz*^*iEC-GOF*^ (**i**) and *Yap/Taz*^*iEC-KO*^ (**j**) mutants. Scale bars, 200 μm. The isolated p-S6^Ser235/236^ signal is shown in grey at the bottom. Arrows indicate the peri-venous region in *Taz*^*iEC-GOF*^ (white) and *Yap/Taz*^*iEC-KO*^ mice (transparent). **k**, Heatmap of solute carrier expression in Ctrl, iYAP^S127A^-, iYAP^S94A/S127A^-, iTAZ^S89^- or iTAZ^S51A/S89A^-expressing HUVECs determined by RNA-seq (*n* = 3 independent samples). **l**,**m**, Immunoblot analysis of SLC7A5 and SLC1A5 in si*YAP/TAZ* (**l**) or si*TEAD1/2/4* (**m**) transfected HUVECs. **n**, SLC7A5 and SLC1A5 protein levels in HUVECs expressing Ctrl, iYAP^S127A^, iTAZ^S89A^, iYAP^S94A/S127A^ or iTAZ^S51A/S89A^. **o**, TEAD-depleted HUVECs fail to induce SLC7A5 and SLC1A5 in response to iYAP^S127A^ or ITAZ^S89A^ overexpression as determined by immunoblotting. **p**, Analysis of endothelial YAP, TAZ and TEAD1 ChIP–seq peaks revealed the TEAD-binding sequence as a highly enriched motif. **q**,**r**, TAZ, YAP and TEAD1 ChIP–seq signals at the *SLC7A5* (**q**) and *SLC3A2* (**r**) genomic loci. RPKMs, reads per kilobase per million mapped reads. Western blot data in **f**–**h** and **l**–**o** are from the respective experiment, processed in parallel and are representative of at least three independent experiments. For **c** and **d**, the Kolmogorov–Smirnov test was used. The unprocessed blots are provided as source data. Source data

**Extended Data Fig. 5:**
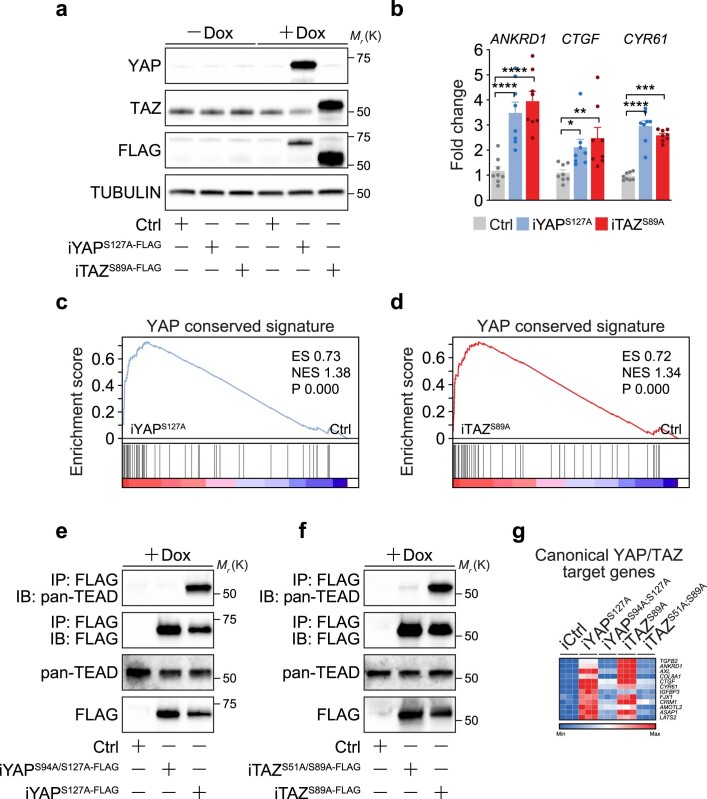
Forced nuclear expression of YAP or TAZ induces canonical YAP/TAZ signaling in ECs. **a**, Immunoblots of HUVECs transduced with doxycycline (Dox)-inducible control (Ctrl), YAP^S127A^ (iYAP^S127A^) and TAZ^S89A^ (iTAZ^S89A^) encoding lentiviruses, showing expression of FLAG-tagged YAP^S127A^ and TAZ^S89A^ upon Dox treatment. Samples were analyzed 48 h after treatment with Dox or vehicle. **b**, RT-qPCR analysis of the canonical YAP/TAZ target genes *ANKRD1, CTGF, and CYR61* in iYAP^S127A^ and iTAZ^S89A^ expressing HUVECs. Expression changes are shown relative to Ctrl (n = 8 independent samples). **c**, **d**, Gene set enrichment analysis (GSEA) showing an enrichment of the YAP-conserved target gene expression signature in the transcriptomes of iYAP^S127A^ (**c**) and iTAZ^S89A^ (**d**) expressing HUVECs. ES, enrichment score; NES, normalized enrichment score. **e**, **f**, FLAG immunoprecipitation studies in HUVECs overexpressing FLAG-tagged iYAP^S127A^ and iYAP^S94A/S127A^ (**e**) or iTAZ^S89A^ and iTAZ^S51A/S89A^ (**f**). The mutation of serine 94 to alanine in YAP and serine 51 to alanine in TAZ disrupts the interaction of (nuclear) YAP and TAZ with TEADs. **g**, Heatmap of mRNA expression changes in HUVECs expressing Ctrl, iYAP^S127A^, iYAP^S94A/S127A^, iTAZ^S89A^ or iTAZ^S51A/S89A^ as determined by RNA-seq. Canonical YAP/TAZ target genes are shown (n = 3 independent samples). Western blot data in **a**, **e** and **f** are from the respective experiment, processed in parallel, and are representative of at least three independent experiments. For **b**, data represent mean ± s.e.m.; two-tailed unpaired t-test. For **c**, **d**, Kolmogorov-Smirnov test. *P < 0.05; **P < 0.01; ***P < 0.001; ****P < 0.0001. The numerical data, unprocessed western blots and *P* values are provided as source data. Source data

To explain how endothelial YAP/TAZ-TEAD promote endothelial proliferation and angiogenic growth, we performed RNA-sequencing (RNA-seq) in HUVECs transduced with doxycycline-inducible lentiviruses encoding for YAP^S127A^, TAZ^S89A^ or GFP as a control (Extended Data Fig. 5a,b). Comparative gene expression analyses at 48 hours of doxycycline treatment revealed that both transcriptional regulators induced profound gene expression changes, with 1,395 genes being up- or downregulated in YAP^S127A^-expressing ECs and 1,410 genes being altered in TAZ^S89A^-transduced cells (log_2_ fold change ≥ 1 and FDR ≤ 0.05) (Fig. 3a,b and Supplementary Table 2). Roughly 78% of the regulated genes overlapped in both transcriptomes (Fig. 3a,b and Supplementary Table 2), indicating that YAP and TAZ can signal redundantly when overexpressed and localized in the nucleus. Among the upregulated genes were prototypical YAP/TAZ targets (for example, *ANKRD1*, *CTGF*, *AXL*, *CYR61*) as well as numerous genes linked to mechanistic target of rapamycin complex 1 (mTORC1) signalling, including genes involved in nutrient uptake (for example, *SLC7A5*, *SLC1A4*, *SLC7A11*, *SLC1A5*, *SLC2A1*), anabolic metabolism (for example, *PSAT1*, *PHGDH*, *ASNS*, *SHMT2*, *HK2*) and cell cycle progression (for example, *BUB1*, *AURKA*,) (Fig. 3c–e and Extended Data Fig. 5b–d). These genes were regulated in a TEAD-dependent manner as YAP/TAZ mutants that are nuclear but fail to interact with TEADs (YAP^S94A;S127A^ and TAZ^S51A;S89A^)^24,25^ did not induce these transcripts (Fig. 3e and Extended Data Fig. 5e–g).

**Extended Data Fig. 6:**
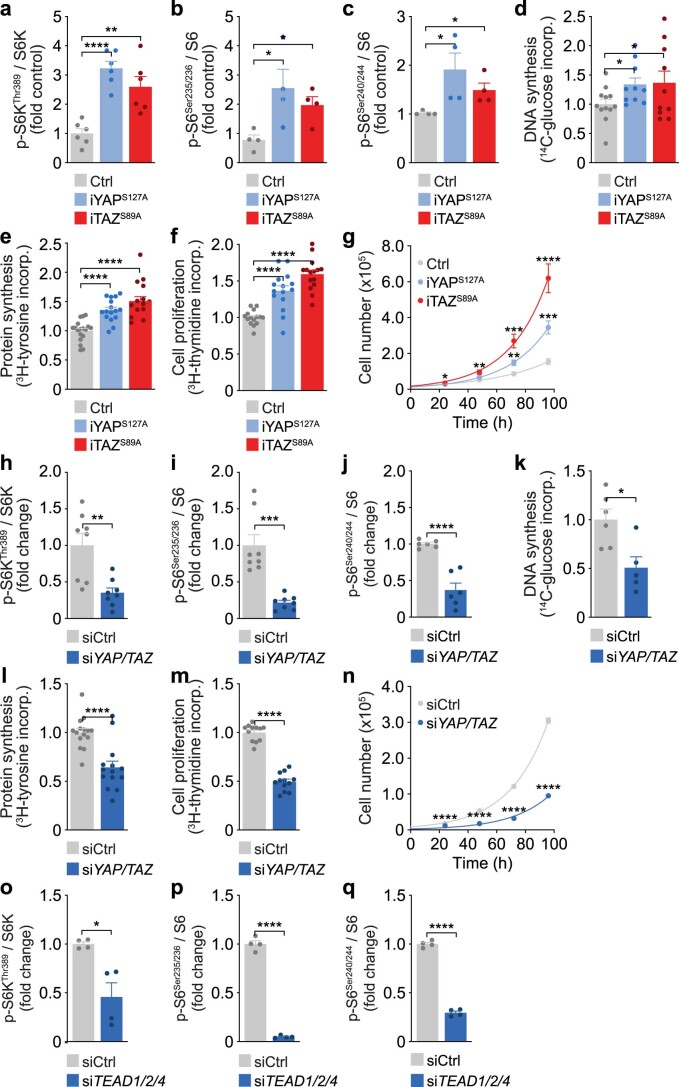
Canonical YAP/TAZ signaling controls mTORC1, anabolism and proliferation in ECs *in vitro*. **a**-**c**, Quantification of S6K (**a**) and S6 (**b**,**c**) phosphorylation in iYAP^S127A^ or iTAZ^S89A^ expressing ECs as determined by immunoblotting (p-S6K^Thr389^: n = 6 independent samples; p-S6^Ser235/236^: n = 4 independent samples; p-S6^Ser240/244^: n = 4 independent samples). **d**, **e**, DNA (**d**) and protein (**e**) synthesis in Ctrl, iYAP^S127A^ and iTAZ^S89A^ HUVECs. Parameters were determined by assessing the incorporation of radiolabeled ^14^C-D-glucose into DNA or ^3^H-tyrosine into protein (DNA synthesis: n = 12 (Ctrl), 10 (iYAP^S127A^) and 11 (iTAZ^S89A^) independent samples; Protein synthesis: n = 18 (Ctrl), 16 (iYAP^S127A^) and 16 (iTAZ^S89A^) independent samples). **f**, Assessment of proliferation in iYAP^S127A^ and iTAZ^S89A^ expressing HUVECs as measured by ^3^H-thymidine DNA incorporation (n = 15 independent samples). **g**, Cell counts over a 96 h period, demonstrating increased cell numbers in iYAP^S127A^ and iTAZ^S89A^ expressing HUVECs when compared to Ctrl (n = 9 independent samples). **h**-**j**, Quantification of S6K (**h**) and S6 (**i**,**j**) phosphorylation in HUVECs transfected with siRNAs targeting *YAP/TAZ* (si*YAP/TAZ*). A pool of non-targeting siRNAs (siCtrl) was used as a control (p-S6K^Thr389^: n = 8 independent samples; p-S6^Ser235/236^: n = 8 independent samples; p-S6^Ser240/244^: n = 6 independent samples). **k**, **l**, Reduction in DNA (**k**) and protein synthesis (**l**) in *YAP/TAZ*-depleted ECs (DNA synthesis: n = 6 (siCtrl) and 5 (si*YAP/TAZ*) independent samples; Protein synthesis: n = 15 (siCtrl) and 14 (si*YAP/TAZ*) independent samples). **m**, Reduced cell proliferation in YAP/TAZ-deficient HUVECs as assessed by ^3^H-thymidine incorporation into DNA (n = 12 independent samples). **n**, Reduced cell counts in si*YAP/TAZ* versus siCtrl HUVECs (n = 9 independent samples). **o-q**, Quantification of S6K (**o**) and S6 (**p**,**q**) phosphorylation in HUVECs transfected with siRNAs targeting *TEAD1*, *TEAD2* and *TEAD4* (si*TEAD1/2/4*). Non-targeting siRNAs (siCtrl) were used as a control (p-S6K^Thr389^: n = 4 independent samples; p-S6^Ser235/236^: n = 4 independent samples; p-S6^Ser240/244^: n = 4 independent samples). For **a – q**, data represent mean ± s.e.m.; two-tailed unpaired t-test. *P < 0.05; **P < 0.01; ***P < 0.001; ****P < 0.0001. The numerical data and *P* values are provided as source data. Source data

mTORC1 is a nutrient-sensitive protein kinase complex that drives cell growth and proliferation through the stimulation of anabolic processes such as protein and DNA synthesis^26–28^. Since the YAP^S127A^- and TAZ^S89A^-induced transcriptional signatures suggested activation of mTORC1 signalling, we asked whether YAP/TAZ regulate mTORC1. To this end, we assessed the phosphorylation state of ribosomal protein S6 kinase (S6K), a substrate of mTORC1, and ribosomal protein S6 (S6), a substrate of S6K1, at mTORC1-sensitive sites (S6K, threonine 389; S6, serine 235/236 and serine 240/244). Compared to controls, YAP^S127A^ and TAZ^S89A^ boosted phosphorylation of these substrates (Fig. 3f and Extended Data Fig. 6a–c). Consistent with an increase in mTORC1 activity, anabolic processes, including DNA and protein synthesis, as well as cell proliferation were also enhanced in these cells (Extended Data Fig. 6d–g). Moreover, small-interfering RNA-mediated depletion of endogenous YAP/TAZ (si*YAP/TAZ*) or TEAD1, TEAD2 and TEAD4 (si*TEAD1/2/4*) suppressed mTORC1 pathway activation in HUVECs (Fig. 3g,h and Extended Data Fig. 6h–q), suggesting that the YAP/TAZ-TEAD transcriptional module is a crucial regulator of endothelial mTORC1.

**Extended Data Fig. 7:**
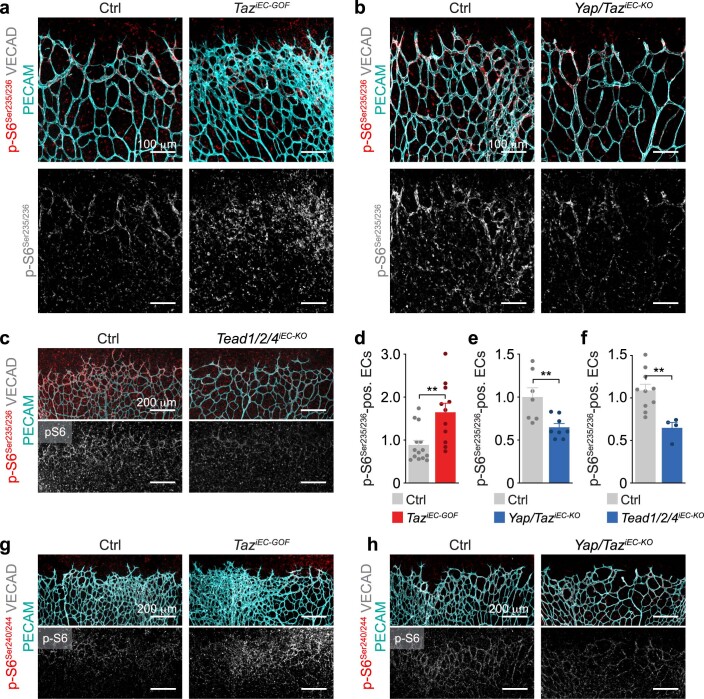
Canonical YAP/TAZ signaling regulates endothelial mTORC1 *in vivo*. **a**,**b**, Higher magnifications images of p-S6^Ser235/236^, VE-cadherin (VECAD), and PECAM immunostainings in P6 retinas of Ctrl, *Taz*^*iEC-GOF*^ (**a**) and *Yap/Taz*^*iEC-KO*^ (**b**) mutants. The isolated p-S6^Ser235/236^ signal is shown in grey at the bottom of the panels. **c**, Immunolabeling of p-S6^Ser235/236^, VECAD, and PECAM in P6 retinas of Ctrl and *Tead1/2/4*^*iEC-KO*^ mice. **d**-**f**, Quantification of endothelial p-S6^Ser235/236^ signals in (**d**) *Taz*^*iEC-GOF*^ (n = 14 (Ctrl) and 10 (*Taz*^*iEC-GOF*^) independent samples), (**e**) *Yap/Taz*^*iEC-KO*^ (n = 7 (Ctrl) and 8 (*Yap/Taz*^*iEC-KO*^) independent samples) and (**f**) *Tead1/2/4*^*iEC-KO*^ mutants (n = 10 (Ctrl) and 4 (*Tead1/2/4*^*iEC-KO*^) independent samples). **g**,**h**, p-S6^Ser240/244^, VECAD, and PECAM immunolabelling in P6 Ctrl, *Taz*^*iEC-GOF*^ (**g**) and *Yap/Taz*^*iEC-KO*^ (**h**) mutants. For **d**-**f**, data represent mean ± s.e.m.; two-tailed unpaired t-test. **P < 0.01. The numerical data and *P* values are provided as source data. Source data

**Extended Data Fig. 8:**
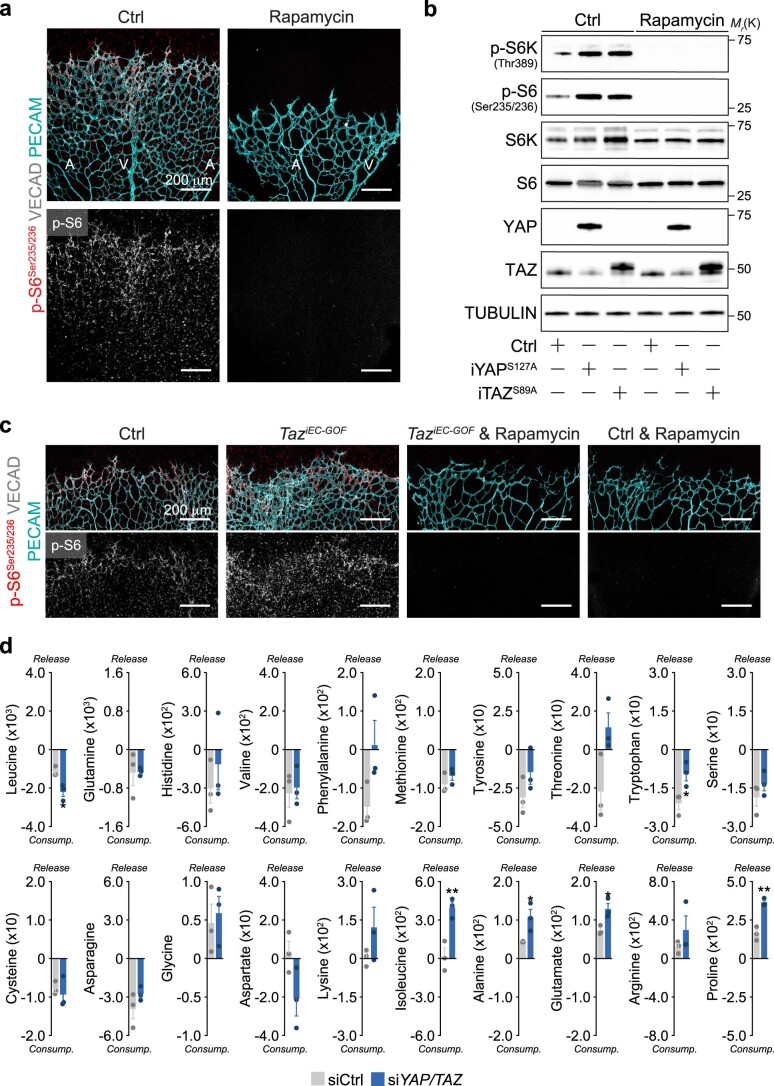
mTOR inhibition prevents TAZ-induced vascular overgrowth. **a**, Immunolabeling of p-S6^Ser235/236^, VECAD and PECAM stained wild-type retinas showing reduced vascular growth and extinguished S6 phosphorylation in mice treated with the mTOR inhibitor rapamycin. Mice were treated with solvent (Ctrl) or rapamycin from P1-P5 and analyzed at P6. The isolated p-S6^Ser235/236^ signal is shown in grey in the lower panels. A, artery; V, vein. **b**, Immunoblot analysis of ECs transduced with doxycycline (Dox)-inducible YAP^S127A^ (iYAP^S127A^), TAZ^S89A^ (iTAZ^S89A^) or control (Ctrl) lentiviruses, and treated with Dox as well as rapamycin or vehicle. **c**, Confocal images showing p-S6^Ser235/236^, VECAD and PECAM stained P6 retinas in Ctrl and *Taz*^*iEC-GOF*^ mice after intraperitoneal administration of vehicle or rapamycin from P1 to P5. Mice were also injected with 4OHT (P1 to P4) to induce *Cre*-mediated recombination of the *Taz*^*GOF*^ allele. The isolated p-S6^Ser235/236^ signal is shown in grey in the lower panels. **d**, Analysis of amino acid consumption and release in control (siCtrl) and YAP/TAZ-deficient (si*YAP/TAZ*) HUVECs as determined by LC-MS/MS (n = 3 independent samples). Western blot data in **b** are from the respective experiment, processed in parallel, and are representative of at least three independent experiments. For **d**, data represent mean ± s.e.m.; two-tailed unpaired t-test, *P < 0.05. **P < 0.01. The numerical data, unprocessed western blots and *P* values are provided as source data. Source data

To confirm these observations in vivo, we labelled P6 mouse retinas with two different S6 antibodies recognizing distinct mTORC1-dependent phosphorylation sites (serine 235/236 → p-S6^Ser235/236^ and serine 240/244 → p-S6^Ser240/244^). The retinas were also labelled with PECAM and vascular endothelial cadherin (VECAD) antibodies to identify ECs. Both p-S6 antibodies gave similar results; in controls, the endothelial p-S6 signal was strongly enriched at sites of active angiogenic growth, particularly at the vascular front and the peri-venous region (Fig. 3i,j and Extended Data Figs. 7a,b and 8a). This labelling pattern was diminished in both *Yap/Taz*^*iEC-KO*^ and *Tead1/2/4*^*iEC-KO*^ mice, yet intensified in *Taz*^*iEC-GOF*^ mutants (Fig. 3i,j and Extended Data Fig. 7a–h). Crucially, the mTOR inhibitor rapamycin extinguished the retinal p-S6 signal (Extended Data Fig. 8a,b), demonstrating the specificity of the antibody labelling. Moreover, rapamycin treatment prevented the overgrowth of ECs in the *Taz*^*S89A*^ mutant (Extended Data Fig. 8c). Together, these results indicate not only a critical role of YAP/TAZ-TEAD in the regulation of endothelial mTORC1 but also of mTORC1 as an effector of the YAP/TAZ-TEAD signalling network.

To decipher how YAP/TAZ-TEAD regulate mTORC1, we searched for YAP/TAZ-induced expression changes in the mTORC1 pathway. YAP^S127A^ and TAZ^S89A^ did not affect the transcripts of the mTORC1 complex *MTOR*, *MLST8*, *AKT1S1* and *RPTOR* nor of its downstream effectors (Supplementary Table 2). Instead, both cofactors induced the transcription of several cell-surface transporters involved in the regulation of mTORC1 by nutrients (Fig. 3k). In particular, a cluster of amino acid transporters was upregulated, including solute carrier family member (SLC) 7A5 (*SLC7A5*), *SLC38A5*, *SLC7A1*, *SLC1A5* and *SLC3A2*, which have been shown to promote mTORC1 signalling^29,30^ (Fig. 3k). Notably, SLC7A5 and SLC3A2 form a heterodimeric amino acid transporter for large neutral amino acids^31^ that cooperates with SLC1A5 to activate mTORC1 (refs. ^29,30^), suggesting a highly coordinated transcriptional response.

We validated the regulation of some of these genes in YAP/TAZ-deficient ECs. Compared to controls, si*YAP/TAZ*-transfected HUVECs expressed lower protein levels of SLC7A5 and SLC1A5, which correlated with a reduced ability to consume several amino acids such as tryptophan, threonine and phenylalanine (Fig. 3l and Extended Data Fig. 8d). The regulation of SLC7A5 and SLC1A5 relied on TEADs because HUVECs overexpressing the TEAD-binding-deficient YAP/TAZ mutants (YAP^S94A;S127A^ or TAZ^S51A;S89A^, respectively) or lacking TEAD1/2/4 failed to upregulate these transporters (Fig. 3k,m–o); findings that are consistent with previous reports in cancer cells^32–34^.

**Extended Data Fig. 9:**
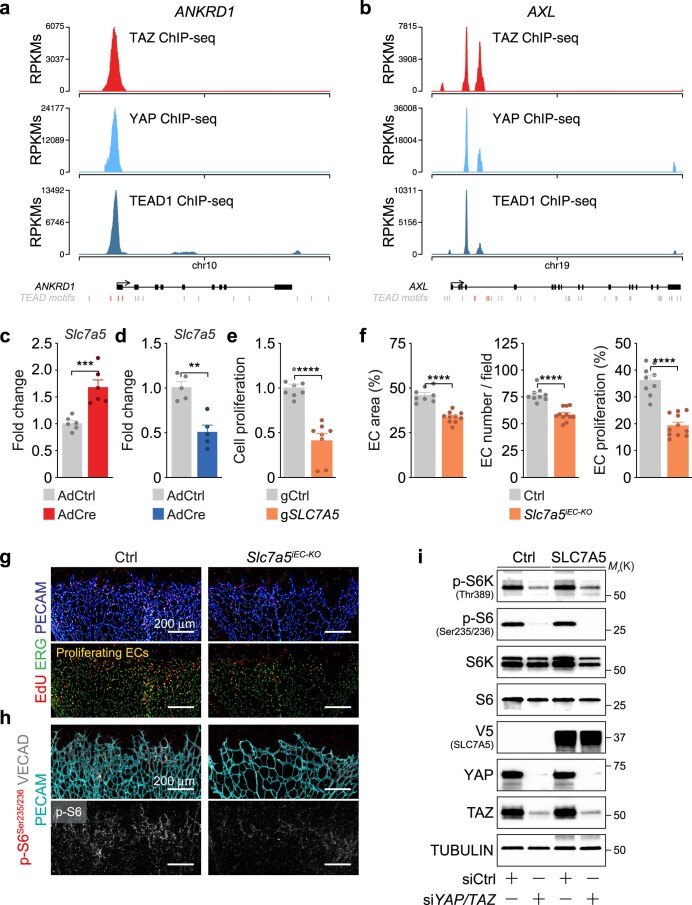
Characterization of direct YAP/TAZ-TEAD target genes in ECs. **a**,**b**, ChIP-seq signals of TAZ, YAP and TEAD1 at the genomic loci of the canonical target genes *ANKRD1* (**a**) and *AXL* (**b**). ChIP-seq signals are represented as reads per kilobase per million mapped reads (RPKMs). **c**,**d**, *Slc7a5* expression in ECs isolated from the lungs of *Rosa26-Taz*^*S89A fl/fl*^ (**c**) or *Yap*^*fl/fl*^*;Taz*^*fl/fl*^ (**d**) mice followed by transduction with control (AdCtrl) or Cre-encoding (AdCre) adenoviruses. *Slc7a5* transcript levels were determined by RT-qPCR (**c**, n = 6 independent samples; **d**, n = 6 independent samples). **e**, Reduced cell proliferation in *SLC7A5*-deficient HUVECs (g*SLC7A5*) as measured by ^3^H-thymidine DNA incorporation (n = 8 independent samples). Values are represented as fold change relative to control (gCtrl). **f**, Quantification of vascular parameters in Ctrl and *Slc7a5*^*iEC-KO*^ mice as indicated (EC area: n = 8 (Ctrl) and 10 (*Slc7a5*^*iEC-KO*^) independent samples; EC number / field: n = 9 (Ctrl) and 11 (*Slc7a5*^*iEC-KO*^) independent samples; EC proliferation: n = 9 (Ctrl) and 11 (*Slc7a5*^*iEC-KO*^) independent samples). **g**, Immunofluorescence staining for EdU, ERG and PECAM in P6 Ctrl and *Slc7a5*^*iEC-KO*^ retinas. **h**, Confocal images of VECAD, p-S6^Ser235/236^ and PECAM stained P6 retinas of Ctrl and *Slc7a5*^*iEC-KO*^ mice, suggesting reduced mTORC1 signaling in *Slc7a5* mutants. The isolated p-S6^Ser235/236^ signal is shown in grey at the bottom of the panel. **i**, Immunoblots of HUVECs transduced with Ctrl or SLC7A5 encoding lentivirus, showing that overexpression of SLC7A5 is insufficient to restore mTORC1 activity in *YAP/TAZ*-depleted HUVECs (si*YAP/TAZ*). Western blot data in **i** are from the respective experiment, processed in parallel, and are representative of at least three independent experiments. For **c**, **d**, **e**-**f**, data represent mean ± s.e.m.; two-tailed unpaired t-test. **P < 0.01; ***P < 0.01; ****P < 0.0001. The numerical data, unprocessed western blots and *P* values are provided as source data. Source data

To further assess whether the regulated transporters are direct YAP/TAZ-TEAD target genes, we performed chromatin immunoprecipitation-sequencing (ChIP–seq) studies. We found that endogenous TAZ bound to the genomic regions of canonical YAP/TAZ targets (for example, *ANKRD1*, *AXL*, *CTGF*, *CYR61*) as well as to several of the regulated transporters, including *SLC7A5*, *SLC3A2*, *SLC1A5*, *SLC7A1* and *SLC7A11* (Fig. 3q,r, Extended Data Fig. 9a,b and Supplementary Table 3). The TEAD consensus sequence was highly enriched in the TAZ ChIP–seq peaks (Fig. 3p), suggesting a critical role for TEADs in recruiting TAZ to chromatin^35^. To confirm this, we performed ChIP–seq for TEAD1, the most abundant TEAD family member in HUVECs (Fig. 2f), and found that TAZ peaks overlapped with TEAD1 peaks. Genes occupied by TAZ and TEAD1 comprised not only canonical targets (for example, *ANKRD1*, *AXL*, *CTGF* and *CYR61*) but also nutrient transporters such as *SLC7A5*, *SLC3A2*, *SLC7A1*, *SLC7A11* (Fig. 3p–r, Extended Data Fig. 9a,b and Supplementary Table 3). Similar results were also obtained for endogenous YAP, although fewer candidate genes were detected (Fig. 3p–r, Extended Data Fig. 9a,b and Supplementary Table 3). Together, these data indicate a model whereby YAP/TAZ-TEAD orchestrate a transcriptional program that facilitates the transport of amino acids and other essential nutrients, thereby enabling mTORC1 pathway activation.

**Fig. 4:**
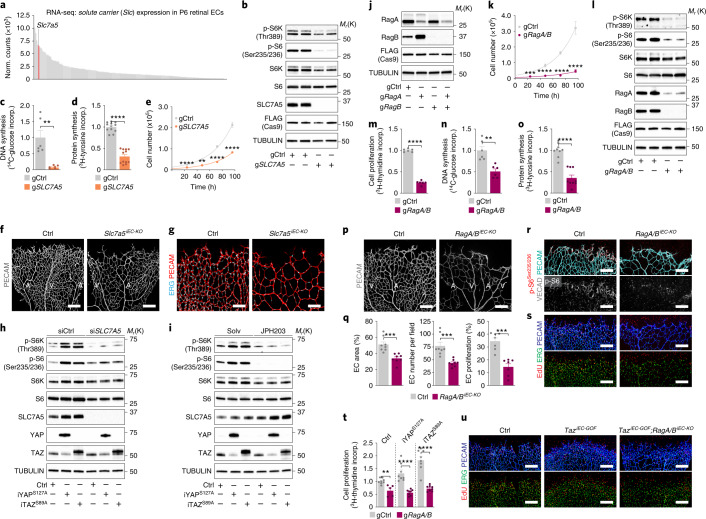
Nutrient-mediated mTORC1 signalling is critical for YAP/TAZ-induced vascular growth. **a**, RNA-seq analysis of solute carrier expression in P6 mouse retinal ECs (*n* = 4 independent samples). **b**, S6K and S6 phosphorylation in control (gCtrl) and SLC7A5-depleted (g*SLC7A5*) HUVECs. Cells were generated by CRISPR–Cas9. **c**,**d**, DNA (**c**) and protein (**d**) synthesis in gCtrl and g*SLC7A5* ECs (DNA synthesis, *n* = 6 independent samples; protein synthesis: *n* = 12 independent samples; incorp., incorporation). **e**, Cell numbers in gCtrl and g*SLC7A5* HUVECs (*n* = 6 independent samples). **f**,**g**, PECAM (**f**) or ERG and PECAM (**g**) labelled P6 retinas of Ctrl (*Slc7a5*^*fl/fl*^) and *Slc7a5*^*iEC-KO*^ (*Pdgfb-creERT2;Slc7a5*^*fl/fl*^*)* mice. A, artery; V, vein. Scale bar **f**, 200 μm; **g**, 100 μm. **h**,**i**, S6K and S6 phosphorylation in Ctrl, iYAP^S127A^ and iTAZ^S89A^ HUVECs, in which SLC7A5 was inactivated by siRNA (si*SLC7A5*) (**h**) or the inhibitor JPH203 (**i**). **j**, RagA/B immunoblots in g*RagA*, g*RagB* and g*RagA/B* HUVECs. **k**, Cell numbers in gCtrl and g*RagA/B* HUVECs (*n* = 6 independent samples). **l**, Analysis of mTORC1 activity markers in RagA/B-depleted HUVECs. **m**, Proliferation is compromised in RagA/B-deficient HUVECs (*n* = 6 independent samples). **n**,**o**, Diminished anabolism in RagA/B-deficient HUVECs (DNA synthesis (**n**): *n* = 6 independent samples; protein synthesis (**o**): *n* = 9 independent samples). **p**, PECAM immunolabelling in P6 Ctrl (*RagA*^*fl/fl*^*;RagB*^*fl/fl*^) and *RagA/B*^*iEC-KO*^ (*Pdgfb-creERT2;RagA*^*fl/fl*^*;RagB*^*fl/fl*^) mice. Scale bar, 200 μm. **q**, Vascular parameters in Ctrl and *RagA/B*^*iEC-KO*^ mice (EC area, *n* = 8 (Ctrl) and 8 (*RagA/B*^*iEC-KO*^) independent samples; EC number/field, *n* = 8 (Ctrl) and 8 (*RagA/B*^*iEC-KO*^) independent samples; EC proliferation, *n* = 7 (Ctrl) and 7 (*RagA/B*^*iEC-KO*^) independent samples). **r**, p-S6^Ser235/236^, VECAD and PECAM labelling of P6 retinas from Ctrl and *RagA/B*^*iEC-KO*^ mice. **s**, Images of P6 Ctrl and *RagA/B*^*iEC-KO*^ retinas labelled for EdU, ERG and PECAM. Scale bars in **r**,**s**, 200 μm. **t**, Proliferation in Ctrl, iYAP^S127A^ and iTAZ^S89A^-ECs subjected to simultaneous depletion of RagA/B (*n* = 8 independent samples). **u**, Images of EdU, ERG and PECAM-labelled P6 retinas in Ctrl, *Taz*^*iEC-GOF*^ *and Taz*^*iEC-GOF*^*;RagA/B*^*iEC-KO*^ mice. Scale bars, 200 μm. Immunoblotting data in **b**,**h**–**j**,**l** are representative of at least three independent experiments. For **c**–**e**,**k**,**m**–**o**,**q** and **t**, data represent mean ± s.e.m.; two-tailed unpaired *t*-test. **P < 0.01; ***P < 0.001; ****P < 0.0001. Numerical data, unprocessed blots and *P* values are provided as source data. Source data

**Extended Data Fig. 10:**
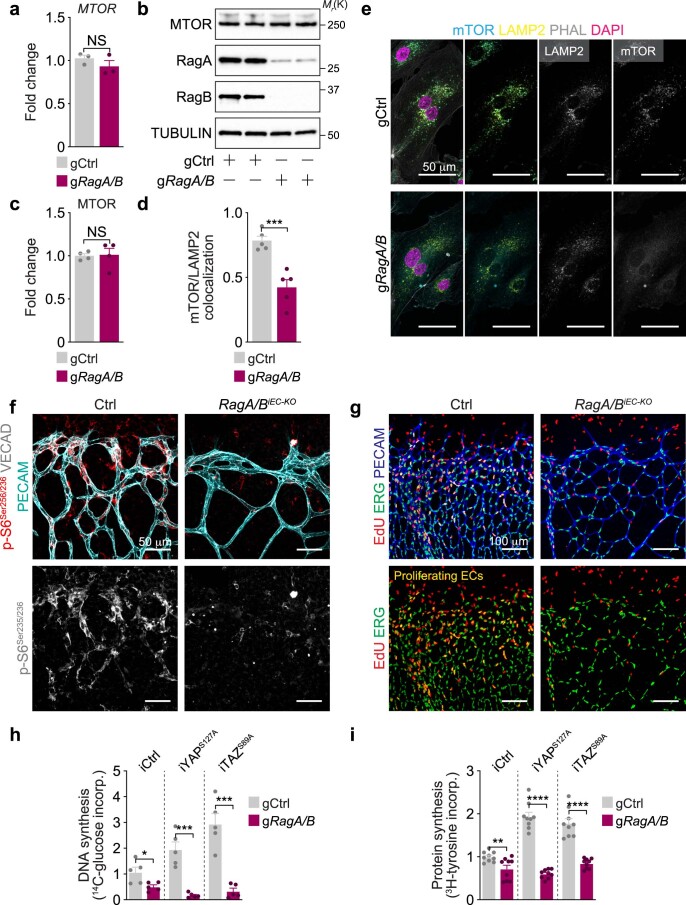
Suppression of endothelial RAG GTPase signaling inhibits mTORC1 and proliferation in ECs. **a**, RT-qPCR analysis in gCtrl and g*RagA/B* HUVECs, showing that RagA/B deficiency does not alter *MTOR* transcript levels (n = 3 independent samples). **b**, **c**, Immunoblot analysis (**b**) and quantification (**c**) of mTOR protein levels in control (gCtrl) or in *RagA/B*-depleted (g*RagA/B*) HUVECs, confirming the RT-qPCR analysis (n = 4 independent samples). **d**, Analysis of mTOR/LAMP2 co-localization in RagA/B-deficient ECs as assessed by immunofluorescence imaging (n = 5 independent samples). **e**, Immunofluorescence images of gCtrl and g*RagA/B* HUVECs stained for the lysosomal protein LAMP2, mTOR, phalloidin (PHAL) and DAPI, showing reduced mTOR/LAMP2 co-localization in g*RagA/B* ECs when compared to gCtrl. **f**, Higher magnification images of P6 Ctrl and *RagA/B*^*iEC-KO*^ mutant mice labelled for p-S6^Ser235/236^, VECAD and PECAM. The images in the lower panel show the isolated p-S6^Ser235/236^ signal in grey. **g**, Higher magnification confocal images of P6 Ctrl and *RagA/B*^*iEC-KO*^ retinas labelled for EdU, ERG and PECAM demonstrating a reduced number of proliferating ECs in the mutants. **h**, **i**, Suppression of DNA (**h**) and protein (**i**) synthesis in Ctrl, iYAP^S127A^ and iTAZ^S89A^ HUVECs subjected to simultaneous depletion of *RagA/B*. DNA synthesis was measured by assessing the incorporation of ^14^C-glucose into DNA (n = 5 independent samples). Protein synthesis was measured by assessing the incorporation of ^3^H-tyrosine into protein (n = 9 independent samples). Western blot data in **b** are from the respective experiment, processed in parallel, and are representative of three independent experiments. For **a**, **c**, **d**, **h** and **i**, data represent mean ± s.e.m.; two-tailed unpaired t-test. *P < 0.05; **P < 0.01; ***P < 0.001; ****P < 0.0001; NS, not significant. The numerical data, unprocessed western blots and *P* values are provided as source data. Source data

To study the functional relevance of the proposed mechanism, we first tested whether altered transporter expression is sufficient to regulate endothelial mTORC1. To this end, we inactivated SLC7A5, one of the most abundant endothelial transporters (Fig. 4a) highly sensitive to YAP/TAZ signalling (Fig. 3k–r and Extended Data Fig. 9c,d). Depletion of SLC7A5 by clustered regulatory interspaced short palindromic repeats (CRISPR)–Cas9 in vitro (g*SLC7A5*) or *Pdgfb-CreERT2*-induced recombination of a floxed allele in vivo (*Slc7a5*^*iEC-KO*^) lowered endothelial mTORC1 signalling and reduced retinal angiogenic growth (Fig. 4b–g and Extended Data Fig. 9e–h). Moreover, inhibiting SLC7A5 function—either by siRNA or the pharmacological inhibitor JPH203—disabled YAP^S127A^ or TAZ^S89A^ to activate endothelial mTORC1 (Fig. 4h,i), underscoring the importance of SLC7A5 for YAP/TAZ signalling responses. Of note, overexpression of SLC7A5 alone was not sufficient to restore mTORC1 activity in YAP/TAZ-depleted HUVECs (Extended Data Fig. 9i), presumably because the levels of SLC7A5’s complex partner SLC3A2 were not restored. To further corroborate the hypothesis that altered nutrient/amino acid acquisition links YAP/TAZ-TEAD to mTORC1, we next inactivated Rag GTPase signalling in ECs. Rags (RagA-D) recruit mTORC1 to the surface of lysosomes when sufficient amino acids are available, thereby allowing full pathway activation^36–38^. To disrupt their signalling and bypass compensatory effects, we inactivated two essential family members, RagA and RagB, simultaneously (Fig. 4j)^39^. CRISPR–Cas9-mediated deletion of *RRAGA* and *RRAGB* (the genes encoding for RagA and RagB, respectively; g*RagA/B*) displaced mTORC1 from the lysosomal surface and suppressed mTORC1 activity (Fig. 4k–o and Extended Data Fig. 10a–e). Consistent with these findings, EC-restricted inactivation of floxed *Rraga* and *Rragb* alleles^39^ in mice (*RagA/B*^*iEC-KO*^) extinguished endothelial p-S6 levels, arrested endothelial proliferation and severely compromised vascular growth (Fig. 4p–s and Extended Data Fig. 10f,g).

Finally, to directly test the requirement of Rag-mediated mTORC1 activity for the anabolic and proliferative functions of endothelial YAP/TAZ, we inactivated Rag signalling in ECs with activated YAP/TAZ. Depletion of RagA/B in HUVECs stalled YAP^S127A^- or TAZ^S89A^-induced anabolism and proliferation (Fig. 4t and Extended Data Fig. 10h,i). Moreover, depletion of RagA/B in mice overexpressing Taz^S89A^ in the endothelium (*Taz*^*iEC-GOF*^*;RagA/B*^*iEC-KO*^) was sufficient to prevent vascular overgrowth in these mutants (Fig. 4u). Collectively, these results establish nutrient-driven mTORC1 signalling as a crucial regulatory pathway in ECs that determines YAP/TAZ-induced vascular expansion, although it seems likely that other RagA/B-independent mechanisms contribute as well.

Our study identifies an essential metabolic link between YAP/TAZ and angiogenic growth, which involves the master regulator of cellular anabolism, mTORC1. We demonstrate that YAP/TAZ form a transcriptional module with TEADs to orchestrate the expression of a cluster of cell-surface transporters, importing amino acids and other metabolic fuels. Signalling by this module supplies ECs with resources for growth and proliferation and enables mTORC1 pathway activation, thereby promoting anabolic processes such as protein and DNA synthesis. By placing nutrient acquisition under the control of YAP/TAZ-TEAD—which integrates mechanical, metabolic and growth factor signals^14,15,18,19^—ECs ensure a coherent angiogenic response that is coupled to the tissue environment. Such coupling is crucial for angiogenic growth as ECs are confronted with changing tissue milieus when forming new vessel branches. The metabolic challenges that arise in these environments might also explain the susceptibility of ECs to perturbed nutrient-regulated mTORC1 signalling, whose disruption causes angiogenic arrest.

Our data indicate that among the regulated cell-surface transporters, SLC7A5 plays a central role. The requirement of this large neutral amino acid carrier for YAP/TAZ-induced mTORC1 activation aligns with previous findings in cancerous cells^32,33^ and highlights the importance of intracellular amino acids for YAP/TAZ-mediated growth responses. Other modes of nutrient acquisition might contribute to the maintenance of sufficient intracellular amino acids levels during angiogenic growth. Macropinocytosis, for instance, is a non-selective endocytic mechanism for bulk ingestion of extracellular macromolecules (proteins)^3^, which has recently been involved in YAP/TAZ signalling^40^ and endothelial biology^41^. These considerations raise the intriguing possibility that YAP/TAZ activity may play a broader role in determining cellular nutrient acquisition strategies and underscore the need to understand these processes in more detail. Delineating the mechanisms that determine how ECs take up, transport and use nutrients will provide new insights not only into vascular (patho-)physiology but also into the role of specific nutrients in instructing vascular growth and function.

## Methods

### Cell culture and treatments

Pooled HUVECs were purchased from Lonza (no. CC-2519) and cultured in endothelial basal medium (Lonza) supplemented with hydrocortisone (1 µg ml^−1^), bovine brain extract (12 µg ml^−1^), gentamicin (50 µg ml^−1^), amphotericin B (50 ng ml^−1^), human recombinant epidermal growth factor (10 ng ml^−1^) and 10% foetal bovine serum (FBS) (Life Technologies). Human aortic ECs (no. CC-2535), human microvascular ECs (no. CC-2813) and human dermal lymphatic ECs (no. CC-2812) were also purchased from Lonza and cultured according to the supplier’s recommendations. Human embryonic kidney cells (HEK293FT) were purchased from Life Technologies (no. R70007) and cultured in DMEM supplemented with 10% FBS (Life Technologies) and gentamicin (50 µg ml^−1^, Lonza). Murine brain and lung ECs were isolated from male and female mice^42^. Briefly, tissues were dissociated using Miltenyi kits (no. 130-098-305; no. 130-095-927) and the gentleMACS Dissociator (Miltenyi, no. 130-096-427). Tissue homogenates were incubated with CD31 microbeads (Miltenyi, no. 130-097-418) and CD31^+^ ECs were purified using LS columns. For brain homogenates, an additional step of myelin depletion (Miltenyi, no. 130-096-731) was performed before incubation with CD31 microbeads. All cells were tested negative for mycoplasma and maintained at 37 °C in a humidified atmosphere with 5% CO_2_. To inhibit mTORC1 or SLC7A5, HUVECs were treated with either 100 nM rapamycin (LC Laboratories, no. LCL-R-5000-50) or 10 µM JPH203 (Selleckchem, no. S8667) using dimethyl sulfoxide as vehicle control.

### RNA interference

HUVECs were transfected with 50 nM of ON-TARGETplus SMARTpool siRNAs (Dharmacon) listed in Supplementary Table 4 using Lipofectamine RNAiMAX (Invitrogen) according to the manufacturer’s recommendations.

### Lentivirus generation and transductions

Human FLAG-YAP^S127A^ (Addgene, no. 27370)^43^ and human FLAG-TAZ^S89A^ (Addgene, no. 24815)^44^, were subcloned into the pLVX-TetOne-Puro vector (Clontech, no. 631847). TEAD-binding-deficient mutants were generated by site-directed mutagenesis of serine 94 to alanine in FLAG-YAP^S127A^ (YAP^S94A/S127A^) and serine 51 to alanine in FLAG-TAZ^S89A^ (TAZ^S51A/S89A^) and mutated DNAs subsequently subcloned into the pLVX-TetOne-Puro. Subcloned plasmids were cotransfected into HEK293FT with lentiviral packaging vectors pMD2.G (Addgene, no. 12259), psPAX2 (Addgene, no. 12260) using Lipofectamine 2000 (Life Technologies). HUVECs were infected with viruses for 16 h in presence of 8 μg ml^−1^ polybrene (Santa Cruz) and selected with 1 μg ml^−1^ puromycin (InvivoGen, no. ant-pr-1). Lentiviral-mediated transgene expression was induced with 100 ng ml^−1^ doxycycline (Sigma, no. D9891) for 48 h before collection.

### CRISPR–Cas9 genome editing of HUVECs

For each target gene, three independent guide RNAs (Supplementary Table 5) were cloned into the plentiCRISPRv2 plasmid (Addgene, no. 52961) and cotransfected with the packaging vectors for lentivirus production as described above. Scramble guide RNA sequences were used as a control (gCtrl).

### Adenoviral transductions

Custom-made adenoviruses to overexpress human FLAG-YAP^S127A^ (AdYAP^S127A^), human FLAG-TAZ^S89A^ (AdTAZ^S89A^) were generated by Vector Biolabs. GFP-encoding adenoviruses were used as controls (AdCtrl, Vector Biolabs, no. 1060). HUVECs were transduced for 4 h in the presence of polybrene (8 µg ml^−1^), washed with Hank’s balanced salt solution and cultured in endothelial basal media with 10% FBS and supplements. Adenoviral transductions of mouse lung ECs isolated from *Rosa-Taz*^*S89A fl/fl*^ and *Yap*^*fl/fl*^*;Taz*^*fl/fl*^ mice were performed with Cre-encoding adenovirus (AdCre, Vector Biolabs, no. 1045) or control (AdCtrl, Vector Biolabs, no. 1300).

### Coimmunoprecipitations

Cells were lysed in IPLS buffer (50 mM Tris-HCL pH7.5, 120 mM NaCl, 0.5 mM EDTA and 0.5% Nonidet P-40) freshly supplemented with protease inhibitor mix (Roche) and 1 mM phenylmethylsulfonyl fluoride^45^. Samples were cleared by centrifugation and protein concentrations determined by the Bradford method. Equal amounts of total lysates were precleared using A/G agarose beads (Santa Cruz Biotechnology, SC-2003) under gentle rotation at 4 °C for 45 min. Epitope tag immunoprecipitations were performed using the anti-FLAG M2 affinity gel beads (Sigma, no. F2426) at 4 °C with gentle rotation for 2 h. Collected beads were washed five times in IPLS buffer and bound proteins eluted in Laemmli sample buffer for subsequent immunoblot analysis. Immunoprecipitations of endogenous YAP and TAZ proteins were performed similarly using antibodies recognizing YAP (Cell Signaling, no. 4912) or TAZ (Santa Cruz Biotechnology, no. SC-48805). Rabbit immunoglobulin G (IgG) (Diagenode, no. C15410206) was used as a control. Precleared lysates were incubated with the antibodies at 4 °C overnight followed by incubation with protein G Sepharose 4 Fast Flow beads (GE Healthcare, no. 17-0618-05) for 2 h. Beads were washed with IPLS buffer five times and eluted proteins subjected to subsequent immunoblotting.

### Mass spectrometry

HUVECs were infected with AdCtrl, AdYAP^S127A^ and AdTAZ^S89A^ and immunoprecipitations performed with FLAG M2 beads 16 h after transduction. Eluates were separated by SDS–PAGE (NuPAGE 4–12% BisTris gel, Invitrogen) and stained with colloidal Protein Staining Solution (Invitrogen). Gel pieces were excised for in-gel digestion using trypsin after reduction and alkylation. After washes with 50% 50 mM NH_4_HCO_3_/50% ethanol for 20 min, gel pieces were dehydrated with 100% ethanol for 10 min and vacuum dried. Samples were reduced with 10 mM dithiothreitol for 45 min at 56 °C and alkylated with 55 mM iodocetamide (BioUltra, Sigma-Aldrich) for 30 min at room temperature in the dark. After washing/dehydration, gel pieces were dehydrated twice with 100% ethanol for 15 min, vacuum dried and digested overnight at 37 °C in 50 μl of digestion buffer containing 12.5 ng μl^−1^ of Sequencing Grade Modified Trypsin (Promega Corp.). Released peptides were extracted once by adding 100 μl of 30% acetonitrile liquid chromatography–mass spectrometry (LC–MS) grade (Thermo Scientific)/3% trifluoroacetic acid (Sigma-Aldrich) in water, and twice by adding 70% acetonitrile, followed by two final extractions with 100% acetonitrile. Extracts were vacuum dried to remove acetonitrile and subsequently acidified with 0.5% trifluoroacetic acid^46^. Peptides were purified by stop and go extraction tips^47^ and analysed by LC–MS using an EASY-nLC chromatograph and a QExactive mass spectrometer (Thermo Fisher Scientific). Peptide/spectrum matching and label-free quantification were performed by the MaxQuant suite of algorithms^48–50^ and data were postprocessed using Perseus^51^.

### RNA-seq analysis and gene set enrichment analysis

RNA was isolated from HUVECs using the miRNeasy Micro Kit (Qiagen) combined with on-column DNase digestion (DNase-Free Set, Qiagen). RNA integrity was verified using LabChip Gx Touch 24 (Perkin Elmer). Then 4 μg of total RNA input was used for Truseq Stranded messenger RNA Library preparation following the low sample protocol (Illumina) and subjected to 1 × 75 bp single end setup sequencing (Illumina NextSeq500) using v.2 chemistry, resulting in minimum of 32 million reads per library. Data quality was assessed using the FastQC v.0.10.1 quality-control tool^52^ for high throughput sequence data. RNA-seq reads were mapped to the human reference genome version hg19 (GRCh37) with STAR software^53^. For gene set enrichment analysis^54,55^, gene set collections from the Molecular Signatures Database (MSigDB) v.4.0 (http://www.broadinstitute.org/gsea/msigdb/) were used. Heat maps were generated using Morpheus, a publicly available program from the Broad Institute (https://software.broadinstitute.org/morpheus/).

### Quantitative PCR with reverse transcription

RNA was isolated using RNeasy Kit (Qiagen) and cDNA synthesized using M-MLV reverse transcriptase (Thermo Fisher). Quantitative PCR with reverse transcription analysis was carried out using the StepOnePlus system (Applied Biosystems). Relative gene expression was calculated with the comparative Ct method and normalized to *ACTB* or *Actb* expression. TaqMan probes used are listed in Supplementary Table 6.

### ChIP

HUVECs were fixed with 1% formaldehyde for 15 min and quenched with 0.125 M glycine. Chromatin was isolated by the addition of lysis buffer, followed by disruption with a dounce homogenizer. Lysates were sonicated and the DNA sheared to an average length of 300–500 bp. Genomic DNA (input) was prepared by treating aliquots of chromatin with RNase, proteinase K and heat for reverse-crosslinking, followed by ethanol precipitation. Pellets were resuspended and the resulting DNA was quantified on a NanoDrop spectrophotometer. Extrapolation to the original chromatin volume allowed quantitation of the total chromatin yield. Sheared chromatin (30 μg) was precleared with protein A agarose beads (Invitrogen). Genomic DNA regions of interest were isolated using ChIP-grade antibodies against YAP1 (Abcam, no. ab52771), TAZ (Sigma, no. HPA007415) or TEAD1 (CST, no. 12292BF). Complexes were washed, eluted from the beads with SDS buffer and subjected to RNase and proteinase K treatment. Crosslinks were reversed by incubation overnight at 65 °C and ChIP DNAs were purified by phenol-chloroform extraction and ethanol precipitation.

### ChIP–seq and analysis

Illumina sequencing libraries were prepared from ChIP and input DNAs by the standard consecutive enzymatic steps of end-polishing, dA-addition, and adaptor ligation. After a final PCR amplification step, the resulting DNA libraries were quantified and sequenced on Illumina’s NextSeq500 (75-nt reads, single end). Reads were aligned to the human genome (hg38) using the Burrows–Wheeler alignment algorithm (default settings). Duplicate reads were removed and only uniquely mapped reads (mapping quality ≥25) were used for further analysis. Alignments were extended in silico at their 3′-ends to a length of 200 bp, which is the average genomic fragment length in the size-selected library, and assigned to 32-nt bins along the genome. The resulting histograms (genomic ‘signal maps’) were stored in bigWig files. Peak locations were determined using the model-based analysis of ChIP–seq (MACS) algorithm (v.2.1.0)^56^ with a cut-off *P* = 1 × 10^−7^. MACS2 peak regions that overlapped with any of the ENCODE blacklist of known false ChIP–seq regions (by a minimum of 1-bp) were removed using a custom-made Perl script (Active Motif). Signal maps and peak locations were used as input data. Binding Motifs were identified with the findMotifsGenome program of the HOMER package^57^ using default parameters and input sequences comprising ±100-bp from the centre of the top 1,000 peaks. All profiles were plotted on a normalized reads-per-million basis. The processed data were plotted and visualized using software of the R project for statistical computing.

### Western blot analysis and antibodies

HUVECs were lysed in RIPA buffer (Sigma, no. R0278) supplemented with 1× EDTA-Free Complete Protease Inhibitor Cocktail (Roche) and 1 mM phenylmethylsulfonyl fluoride^58^. Proteins were resolved by SDS–PAGE using Criterion TGX Precast gels (Bio-Rad) and transferred onto nitrocellulose membranes using the Trans Turbo Blot system (Bio-Rad). Membranes were blocked in 5% BSA or 5% milk + 0.01% Tween-20 in TBS 1× for 1 h at room temperature. Primary antibodies in blocking buffer were incubated overnight at 4 °C. Peroxidase-conjugated secondary antibodies were incubated for 1 h at room temperature. Immunoblots were visualized using Clarity Western ECL kit (Bio-Rad) and the ChemiDoc MP Imaging System (Bio-Rad). Band intensities were quantified using the Image Lab software (Bio-Rad). Antibodies used are listed in Supplementary Table 7.

### Proliferation assays

HUVECs were seeded on six-well plates at 2.5 × 10^4^ cells per well and allowed to attach overnight. Next day (0 h), the total cell number was counted with a hemocytometer. Cell counts were repeated every 24 h and culture medium replaced every 48 h. For [^3^H]-thymidine DNA incorporation^59^, cells were seeded on 24-well plates at 5 × 10^4^ cells per well and allowed to attach overnight. HUVECs were pulsed with cell culture medium containing 1 μCi per ml [^3^H]-thymidine for 6 h before collection. Cells were washed with ice-cold PBS, fixed with 100% ethanol for 15 min at 4 °C and precipitated with 10% trichloroacetic acid for 15 min at 4 °C. After washing three times with Hank’s balanced salt solution, cells were lysed with 0.1 N NaOH for 10 min at room temperature and the amount of [^3^H]-thymidine incorporated into DNA was measured with a Liquid Scintillation Analyser Tri-Carb 2810R (Perkin Elmer). Data were normalized to total protein content and expressed as fold change.

### Metabolic flux assays

To determine protein synthesis, HUVECs were incubated with medium containing 1 μCi per ml [^3^H]-tyrosine (Perkin Elmer) for 6 h. Cells were washed with ice-cold PBS, proteins precipitated with 10% trichloroacetic acid overnight and collected by centrifugation at 21.000*g* for 5 min. The protein pellet was resuspended in 0.5 M NaOH with 0.1% (v/v) Triton X-100 and the amount of [^3^H]-tyrosine incorporated into protein was measured by scintillation counting and subsequently normalized to protein content^60^. Glucose-dependent DNA synthesis was measured by assessing the incorporation of ^14^C into DNA using 2.9 mCi per mmol [U^14^C]-glucose (Perkin Elmer). Incorporation was analysed at 48 h in triplicate and measured by scintillation counting. Counts were normalized to the total amount of DNA per sample. Total DNA was isolated using Trizol.

### Metabolomics and CoRe analysis

HUVECs (2.5 × 10^5^) were plated onto six-well plates and transfected with the indicated siRNAs. The culture medium was replaced 4 h after transfection (*t* = 0) and cells were incubated for additional 36 h. A control plate with culture medium (no cells) was prepared at *t* = 0 and processed in parallel at the experimental end-point. The collected media were centrifuged at 4 °C for 10 min at 21,000*g* and 50 µl of the supernatant was extracted in 750 µl of cold metabolite extraction solution (50% methanol, 30% acetonitrile, 20% water). Extracts were placed for 15 min over dry ice, vortexed and incubated in a Thermomixer (1.400 r.p.m.) at 4 °C for 15 min. Samples were incubated for 1 h at −20 °C and metabolite extracts were cleared by centrifugation at 4 °C for 10 min at 21,000*g*, transferred into autosampler vials and stored at −80 °C until further analysis. Cell culture media extracts from cell cultures were analysed for each condition. Samples were randomized to avoid bias due to machine drift and processed blindly. LC–MS analysis was carried out using a Vanquish Horizon UHPLC system coupled to a QExactive HF mass spectrometer (both Thermo Fisher Scientific). Sample extracts (5 µl) were injected onto a Sequant ZIC-pHILC column (150 × 2.1 mm, 5 µm) and guard column (20 × 2.1 mm, 5 µm, Merck Millipore) kept at 45 °C. The mobile phase was composed of 20 mM ammonium carbonate and 0.1% ammonium hydroxide in water (solvent A) and acetonitrile (solvent B). The mobile phase was composed of 20 mM ammonium carbonate with 0.1% ammonium hydroxide in water (solvent A) and acetonitrile (solvent B). The flow rate was set at 200 μl min^−1^ with the following gradient: 0 min 80% B, 2 min 80% B, 17 min 20% B, 17.1 min 80% B and a hold at 80% B for 5 min (ref. ^61^). The mass spectrometer was operated in full MS and polarity switching mode. The acquired spectra were analysed using XCalibur Qual Browser and XCalibur Quan Browser software (Thermo Fisher Scientific) by referencing to an internal library of compounds. To obtain a relative measure of the metabolite consumption/release (CoRe), the background levels of each metabolite in the media controls were subtracted to the levels measured in the cell-conditioned media samples and adjusted to the average cell number.

### Genetic mouse models and pharmacological treatments

The following published mouse lines were used in this study: *Yap*^*fl/fl*^ (ref. ^20^)., *Taz*^*fl/fl*^ (ref. ^21^), *Tead4*^*fl/fl*^ (ref. ^62^), *Rraga* ^*fl/fl*^ (ref. ^39^), *Rragb*^*fl/fl*^ (ref. ^39^), *Slc7a5*^*fl/fl*^ (refs. ^63,64^) and *Pdgfb-iCreERT2-IRES-EGFP* (*Pdgfb-creERT2*)^65^. For the construction of the Cre-activated *Taz gain-of-function* allele (*Taz*^*GOF*^), a *3xFLAG-TAZ*^*S89A*^-*IRES-nEGFP* sequence preceded by a floxed Neomycin-STOP cassette was knocked into the *Rosa26* locus. Cre-mediated removal of the *STOP* sequence results in *CAG* promoter-driven expression of *3xFLAG-TAZ*^*S89A*^ and nuclear-localized enhanced GFP (*nEGFP*). To generate a *Taz* knock-in reporter mouse (*Taz*^*tag*^), a fusion tag consisting of GFP, FLAG and a biotin-labelling peptide was inserted in-frame upstream of the stop codon of the endogenous *Wwtr1* (*Taz*) locus. The conditional *Tead1* knockout allele was generated by flanking exons 3 to 5 with *loxP* sites, while the straight knockout allele of *Tead2* (*Tead2*^*ko*^) was generated by deleting exons 1 to 4. The conditional *Taz*^*GOF*^ and *Taz*^*tag*^ knock-in alleles were developed together with genOway. All mice were bred on a C57BL/6J genetic background. Floxed mice were crossed to mice expressing the tamoxifen-inducible *Pdgfb* promoter-driven *CreERT2* recombinase. Littermates that were negative for *CreERT2* were used as controls. For the combined inactivation of *Tead1*, *Tead2* and *Tead4*, *Tead1*^*fl/fl*^*;Tead2*^*wt/ko*^ mice (*Tead1* and *Tead2* are both located on chromosome 7) were interbred with *Tead4*^*fl/fl*^ and *Pdgfb-CreERT2* mice. To activate CreERT2, pups were administered 25 μl of 4-hydroxytamoxifen (4OHT; 2 mg ml^−1^) intraperitoneally from P1 to P4. Animals were euthanized and retinas harvested at P6 (Supplementary Table 8). To inhibit mTOR signalling, animals were randomly divided into two groups and injected with vehicle or 2 µg g^−1^ rapamycin (LC Laboratories) from P1 to P5. To detect proliferating cells, pups were administered 25 µl of 5-ethynyl-2′-deoxyuridine (EdU) (6 mg ml^−1^; Invitrogen, A10044) intraperitoneally 3 h before euthanasia. Both male and female animals were used. Animal experiments were performed in accordance with institutional guidelines and protocols approved by the Committee for Animal Rights Protection of the State of Hessen (Regierungspraesidium Darmstadt) with the project numbers B2/1061 and B2/1230.

### Sorting of retinal ECs

Retina pairs from P6 C57BL/6J mice were freshly dissected and digested in 1 ml of DMEM with 2.5 mg ml^−1^ type II collagenase (Sigma C6885) with orbital shaking at 300 r.p.m., 37 °C for 30 min. Homogenates were strained through a 70 µm filter and centrifuged at 300*g*, 10 min at 4 °C. Cell pellets were resuspended in 100 μl of PEB buffer (PBS + 2 mM EDTA + 0.5% BSA) and incubated with CD31-FITC (BD Biosciences, no. 553372) and CD45-PECy7 (Invitrogen, no. 25-0451-82) for 30 min at 4 °C. Viable ECs (CD31^+^/CD45^−^) were sorted on a BD FACSAria III (BD Bioscience) straight into RNA Lysis buffer.

### Immunofluorescence

HUVECs were plated on glass bottom dishes (Mattek) and fixed with 4% paraformaldehyde for 30 min at room temperature. Permeabilization and blocking was performed in 1% BSA, 10% FBS and 0.5% Tween-20 in PBS. Primary antibodies were incubated in blocking buffer at 4 °C overnight. Following washes with PBST (0.1% Tween-20 in PBS), cells were incubated with AlexaFluor-conjugated secondary antibodies (Invitrogen) for 2 h at room temperature and mounted using VectaShield (Vector Laboratories, no. H-100). For mouse retina immunostaining, eyes were fixed in 4% paraformaldehyde on ice for 2.5 h (ref. ^66^). After dissection, retinas were incubated in blocking buffer (3% FBS, 1% BSA, 0.25% Tween-20 and 0.25% Triton X-100 in PBS) for 1 h at room temperature. Primary antibodies were incubated in blocking buffer diluted in PBS (1:1) overnight at 4 °C. After washing in PBST, retinas were incubated with Alexa-conjugated secondary antibodies for 2 h at room temperature, washed and flat-mounted with ProLong Gold (Life Technologies). To detect EdU-labelled DNA, an additional step was performed before mounting using the Click-It EdU kit (Invitrogen, no. C10338). Immunostainings were performed in tissues from littermates and processed under the same conditions except for studies with the *Tazi*^*EC-GOF*^*;RagA/B*^*iEC-KO*^ mice given the very low allele frequency. Images were acquired with a Leica confocal microscope SP8. For the comparisons of phenotypes or signal intensities, setting for laser excitation and detector were kept constant between groups. Volocity (Perkin Elmer), Fiji/ImageJ, Photoshop (Adobe) and Illustrator (Adobe) software were used for image acquisition and processing.

### Quantitative analysis of retinal vasculature

Endothelial coverage was quantified from confocal fields behind the angiogenic front in between arteries and veins using Volocity (Perkin Elmer). Endothelial coverage (EC area) was measured as the ratio of PECAM-positive area to total area of vascularized field (sized 200 × 200 μm^2^). EC proliferation was scored as the ratio of EdU and ERG double-positive cells to the total number of ERG-positive cells per field. All parameters were quantified from at least three vascularized fields per sample. To quantify p-S6 levels in retinal ECs, the absolute intensity of the p-S6 and PECAM double-positive area was quantified using Imaris (Bitplane) and expressed as fold change relative to controls.

### Statistics and reproducibility

For quantitative analyses, a minimum of three biological replicates were analysed. Western blot data are from the respective experiment, processed in parallel and are representative of at least three independent experiments. Images from immunofluorescence studies are representative of the respective phenotype observed in samples from at least three independent experiments/litters. Statistical analyses were performed by unpaired, two-tailed Student’s *t*-test unless indicated otherwise. For all bar graphs, data are represented as mean ± s.e.m. A value of *P* < 0.05 was considered significant. No statistical method was used to predetermine sample size. Calculations were performed using the Prism v.9.0 software (GraphPad Software Inc.). Numerical data and exact *P* values are provided as source data.

### Reporting summary

Further information on research design is available in the Nature Research Reporting Summary linked to this article.

## Supplementary information

Reporting Summary

Supplementary Table 1 Proteins interacting with YAP^S127A^ and TAZ^S89A^ in HUVECs (*n* = 3 independent samples). A two-tailed unpaired *t*-test was used to calculate *P* values.

Supplementary Table 2 Differentially expressed genes in HUVECs expressing YAP^S127A^ and TAZ^S89A^ (*n* = 3 independent samples). The Wald test was used to calculate *P* values.

Supplementary Table 3 Genomic localization of YAP, TAZ and TEAD1 binding sites at the genomic loci of YAP/TAZ-regulated genes.

Supplementary Table 4 Gene-specific siRNAs.

Supplementary Table 5 Gene-specific gRNA sequences used for CRISPR–Cas9 genome editing studies in HUVECs.

Supplementary Table 6 Quantitative PCR with reverse transcription probes.

Supplementary Table 7 Antibodies for western blotting and immunofluorescence.

Supplementary Table 8 Genetic mouse models.

## Data Availability

RNA- and ChIP–seq datasets have been deposited in National Center for Biotechnology Information Gene Expression Omnibus with the accession number GSE163459. The mass spectrometry proteomics data have been deposited to the ProteomeXchange Consortium via the PRIDE partner repository with the dataset identifier PXD026872. Source data are provided with this paper. All other data supporting the findings of this study are available from the corresponding author upon reasonable request.

## Source data

Source Data Fig. 1 Unprocessed blots.
Source Data Fig. 1 Statistical source data.
Source Data Fig. 2 Unprocessed blots.
Source Data Fig. 2 Statistical source data.
Source Data Fig. 3 Unprocessed blots.
Source Data Fig. 4 Unprocessed blots.
Source Data Fig. 4 Statistical source data.
Source Data Extended Data Fig. 1 Statistical source data.
Source Data Extended Data Fig. 2 Unprocessed blots and gels.
Source Data Extended Data Fig. 2 Statistical source data.
Source Data Extended Data Fig. 3 Unprocessed blots.
Source Data Extended Data Fig. 3 Statistical source data.
Source Data Extended Data Fig. 4 Statistical source data.
Source Data Extended Data Fig. 5 Unprocessed blots.
Source Data Extended Data Fig. 5 Statistical source data.
Source Data Extended Data Fig. 6 Statistical source data.
Source Data Extended Data Fig. 7 Statistical source data.
Source Data Extended Data Fig. 8 Unprocessed blots.
Source Data Extended Data Fig. 8 Statistical source data.
Source Data Extended Data Fig. 9 Unprocessed blots.
Source Data Extended Data Fig. 9 Statistical source data.
Source Data Extended Data Fig. 10 Unprocessed blots.
Source Data Extended Data Fig. 10 Statistical source data.

## Extended data

are available for this paper at 10.1038/s42255-022-00584-y.
