# Supplementary material for: A YAP/TAZ-TEAD signalling module links endothelial nutrient acquisition to angiogenic growth
Source: Nat Metab. 2022 Jun 20;4(6):672–82. doi: 10.1038/s42255-022-00584-y (PMC9236904; doi:10.1038/s42255-022-00584-y)
Supplement: Supplementary file 1 — Reporting Summary [file 42255_2022_584_MOESM1_ESM.pdf]

nature research

Corresponding author(s): Michael Potente

Last updated by author(s): May 2, 2022

Reporting Summary

Nature Research wishes to improve the reproducibility of the work that we publish. This form provides structure for consistency and transparency in reporting. For further information on Nature Research policies, see our Editorial Policies and the Editorial Policy Checklist.

Statistics

For all statistical analyses, confirm that the following items are present in the figure legend, table legend, main text, or Methods section.

n/a	Confirmed	
☐	☒	The exact sample size (*n*) for each experimental group/condition, given as a discrete number and unit of measurement
☐	☒	A statement on whether measurements were taken from distinct samples or whether the same sample was measured repeatedly
☐	☒	The statistical test(s) used AND whether they are one- or two-sided<br>*Only common tests should be described solely by name; describe more complex techniques in the Methods section.*
☒	☐	A description of all covariates tested
☒	☐	A description of any assumptions or corrections, such as tests of normality and adjustment for multiple comparisons
☐	☒	A full description of the statistical parameters including central tendency (e.g. means) or other basic estimates (e.g. regression coefficient) AND variation (e.g. standard deviation) or associated estimates of uncertainty (e.g. confidence intervals)
☐	☒	For null hypothesis testing, the test statistic (e.g. *F*, *t*, *r*) with confidence intervals, effect sizes, degrees of freedom and *P* value noted<br>*Give P values as exact values whenever suitable.*
☒	☐	For Bayesian analysis, information on the choice of priors and Markov chain Monte Carlo settings
☒	☐	For hierarchical and complex designs, identification of the appropriate level for tests and full reporting of outcomes
☒	☐	Estimates of effect sizes (e.g. Cohen's *d*, Pearson's *r*), indicating how they were calculated

Our web collection on statistics for biologists contains articles on many of the points above.

Software and code

Policy information about availability of computer code

Data collection	qRT-PCR: StepOnePlus real-time PCR system (Applied Biosystems)<br>RNA- and ChIP-seq: NextSeq500 (Illumina)<br>Imaging: SP8 confocal microscope (Leica) and IncuCyte System (Essen BioScience)<br>Western-blot: Chemidoc MP Imaging System (Bio-Rad)<br>Scintillation counting: Liquid Scintillation Analyzer Tri-Carb 2810R (Perkin Elmer)
Data analysis	Graphs: GrahPad Prism v8.0<br>RNA- and ChIP-Seq: R project; MACS; BWA (v0.7.12); bcl2fastq2 (v2.20); Samtools (v0.1.19); BEDtools (v2.25.0); wigToBigWig (v4)<br>Image analysis: Image Lab 5.1(Biorad), ImageJ/FIJI (v2.0.0-rc-69/1.52p), Adobe Photoshop 2020 (v21.2.3), Adobe Illustrator 2020 (v24.3) and Volocity 6.3 (Perkin Elmer)

For manuscripts utilizing custom algorithms or software that are central to the research but not yet described in published literature, software must be made available to editors and reviewers. We strongly encourage code deposition in a community repository (e.g. GitHub). See the Nature Research guidelines for submitting code & software for further information.

Data

Policy information about availability of data

All manuscripts must include a data availability statement. This statement should provide the following information, where applicable:

- Accession codes, unique identifiers, or web links for publicly available datasets
- A list of figures that have associated raw data
- A description of any restrictions on data availability

The data supporting the findings of this study are available within the paper. Genome wide transcriptome data have been deposited in NCBI Gene Expression

Omnibus under the accession numbers GSE163456 and GSE199858. ChIP-seq data have been deposited under the accession number GSE163458. The mass spectrometry proteomics data have been deposited to the ProteomeXchange Consortium via the PRIDE partner repository with the dataset identifier PXD026872.

The following publicly available databases were used:
GSEA pathway analysis: MsigDB (https://www.gsea-msigdb.org/gsea/msigdb);
gRNA design: Genetic Perturbation Platform (https://portals.broadinstitute.org/gpp/public/);

Any additional information required to interpret, replicate or build upon the findings of this study are available from the corresponding author upon reasonable request.

Field-specific reporting

Please select the one below that is the best fit for your research. If you are not sure, read the appropriate sections before making your selection.

☒ Life sciences ☐ Behavioural & social sciences ☐ Ecological, evolutionary & environmental sciences

For a reference copy of the document with all sections, see nature.com/documents/nr-reporting-summary-flat.pdf

Life sciences study design

All studies must disclose on these points even when the disclosure is negative.

Sample size	Sample size for each experiment is indicated in the figure legend. Sample sizes were selected on the basis of published protocols (Pitulesco et al., Nat. Protocols, 2010) and previous experiments (Wilhelm et al., Nature,2016; Lim et al., Science, 2019; Luo et al., Nature, 2020). No statistical methods were used to predetermine sample size. Images are representative of at least three independent experiments in mice or cells of the same treatment group or genotype. Western blot data were from the respective experiment, processed in parallel, and are representative of at least three independent experiments.
Data exclusions	Mice with clear developmental delay or unexpected death were excluded from analysis.
Replication	All experimental findings were reproduced in multiple independent experiments. For each panel, the number of independent experiments or biological replicates is indicated in the figure legend.
Randomization	No statistical methods were used for randomization. For mouse experiments, mice of the requisite genotype were randomly selected to receive the drug or vehicle.
Blinding	Investigators were not blinded since mice and cells were selected for analysis based on their genotype/treatment.

Reporting for specific materials, systems and methods

We require information from authors about some types of materials, experimental systems and methods used in many studies. Here, indicate whether each material, system or method listed is relevant to your study. If you are not sure if a list item applies to your research, read the appropriate section before selecting a response.

Materials & experimental systems

n/a	Involved in the study	
☐	☒	Antibodies
☐	☒	Eukaryotic cell lines
☒	☐	Palaeontology and archaeology
☐	☒	Animals and other organisms
☒	☐	Human research participants
☒	☐	Clinical data
☒	☐	Dual use research of concern

Methods

n/a	Involved in the study	
☐	☒	ChIP-seq
☒	☐	Flow cytometry
☒	☐	MRI-based neuroimaging

Antibodies

Antibodies used

Primary antibodies for Western blot analysis:
ASCT2 (Cell Signaling Technology, #5345, 1:1000, rabbit)
FLAG M2 (Sigma, #F-3165, 1:4000, mouse)
GAPDH (Cell Signaling Technology, #2118, 1:5000, rabbit)
GFP (Cell Signaling Technology, #2555, 1:5000, rabbit)
LAT1 (Cell Signaling Technology, #5347, 1:1000, rabbit)
mTOR (Cell Signaling Technology,#2983, 1:1000, rabbit)
Pan-TEAD (Cell Signaling Technology, #13295, 1:1000, rabbit)
Phospho-4EB-P1 (Ser65) (Cell Signaling Technology, #9456, 1:5000, rabbit)

Phospho-p70 S6 Kinase (Thr389) (Cell Signaling Technology, #9205, 1:1000, rabbit)
p70 S6 Kinase (Cell Signaling Technology, #9202, 1:1000, rabbit)
Phospho-S6 (Ser 235/236) Ribosomal protein (Cell Signaling Technology, #4857, 1:5000, rabbit)
Phospho-S6 (Ser240/244) ribosomal protein (Cell Signaling Technology, #2215, 1:5000, rabbit)
RAGA (Cell Signaling Technology, #4357, 1:1000, rabbit),
RAGB (Cell Signaling Technology, #8150, 1:1000, rabbit)
S6-Ribosomal protein (Cell Signaling Technology, #2217, 1:5000, rabbit)
TAZ (Cell Signaling Technology, #4883, 1:2000, rabbit)
TEAD4 (Santa Cruz, #sc-101184, 1:1000, mouse)
TUBULIN (Cell Signaling Technology, #2148, 1:5000, rabbit)
VEGFR2 (Cell Signaling Technology, #2479, 1:1000, rabbit)
YAP (Cell Signaling Technology, #4912, 1:1000, rabbit)
YAP/TAZ (Cell Signaling Technology, #8418, 1:1000, rabbit)
4E-BP1(Cell Signaling Technology, #9644, 1:5000).

Secondary antibodies for Western blot analysis:
anti-rabbit HRP-conjugated (Jackson Immuno Research Labs, 111-035-008, 1:5000, goat)
anti-mouse HRP-conjugated (Jackson Immuno Research Labs, 315-035-003, 1:5000, rabbit)
TrueBlot anti rabbit IgG (Rockland Immunochemicals, #18-8816-33, 1:2000, goat)

Primary antibodies for immunohistochemical analysis:
ERG (Abcam, #ab92513, 1:400, rabbit)
GFP (Acris, #R1091P, 1:500, goat)
LAMP2 (Abcam, #Ab25631, 1:100, mouse)
mTOR (Cell Signaling Technology, #2983, 1:100, rabbit)
Phospho-S6 (Ser235/236) Ribosomal protein (Cell Signaling Technology, #4857, 1:100, rabbit)
Phospho-S6 (Ser240/244) ribosomal protein (Cell Signaling Technology, #2215, 1:5000, rabbit)
PECAM-1/CD31 (R&D Biosystems, #AF3628, 1:200, goat)
TAZ (Sigma, #HPA007415, 1:200, rabbit)
VECAD (BD Biosciences, #555289, 1:200, rat)

Secondary antibodies for immunohistochemical analysis:
anti-goat IgG, Alexa Fluor 647 (Life Technology, #A21447, donkey)
anti-goat IgG, Alexa Fluor 555 (Life Technology, #A21432, donkey)
anti-goat IgG, Alexa Fluor 488 (Life Technology, #A11055, donkey)
anti-rabbit IgG, Alexa Fluor 488 (Life Technology, #A21206, donkey)
anti-rabbit IgG, Alexa Fluor 555 (Life Technology, #A31572, donkey)
anti-rat IgG, Alexa Fluor 488 (Life Technology, #A21208, donkey)

Validation

All antibodies used in this manuscript were obtained from the indicated commercial vendors and have been validated by the respective manufacturer, as described in their website. All antibodies were used in multiple experiments to detect the indicated target protein giving results according to the expected molecular weight, tissue expression pattern and subcellular localization.

Eukaryotic cell lines

Policy information about cell lines

Cell line source(s)

Pooled human umbilical vein endothelial cells (HUVECs; #CC-2519), human arterial endothelial cells (HAECs; #CC-2535), human microvascular endothelial cells (HMVECs, #CC-2813) and human dermal lymphatic endothelial cells (HDLECs, #CC-2812) were obtained from Lonza.
Human embryonic kidney cells (HEK293FT) were purchased from LifeTechnologies (#R70007).

Authentication

None of the cell lines were authenticated by us.

Mycoplasma contamination

Cells were tested negative for mycoplasma.

Commonly misidentified lines
(See ICLAC register)

No commonly misidentified cell lines were used.

Animals and other organisms

Policy information about studies involving animals; ARRIVE guidelines recommended for reporting animal research

Laboratory animals

All mice used were on a C57BL/6 genetic background and were kept in environmental conditions of 45–65% relative humidity, temperatures of 20-24°C and a 12h-12 h light-dark cycle, with food and water 'ad libitum'. For the construction of the TAZ gain-of-function allele, 3xFlag-TAZS89A-IRES-nEGFP sequence preceded by a floxed Neomycin-STOP cassette was knocked into the Rosa26 locus. To generate a Taz reporter mouse, a fusion tag consisting of GFP-FLAG-AVI was knocked-in into C terminal of murine Wwtr1 endogenous locus located upstream of the stop codon (exon 7). Both alleles were developed together with genOway. The conditional Tead1 knockout allele was generated by flanking exons 3 to 5 with loxP sites, while the straight knockout allele of Tead2 (Tead2 -/-) was generated by deleting exons 1 to 4. Floxed mice were crossed to transgenic mice expressing the tamoxifen-inducible, Pdgfb promoter-driven creERT2, and analysis was performed on post-natal day 6. For Rapamycin treatment on wild type mice, animals were randomly divided into control or Rapamycin group and administered solvent (vehicle) or Rapamycin from post-natal day 1 to day 5. For Rapamycin experiments performed on TaziEC-GOF mice, all pups were additionally injected daily with 4-hydroxy-tamoxifen

	from post-natal day 1 to 4 followed by retinal vasculature analysis at post-natal day 6.
Wild animals	Wild animals were not used in this study.
Field-collected samples	The study did not involve animals from the field.
Ethics oversight	Experiments involving animals were conducted in accordance with institutional guidelines and laws, following protocols approved by local animal ethics committees and authorities (Regierungspraesidium Darmstadt).

Note that full information on the approval of the study protocol must also be provided in the manuscript.

ChIP-seq

Data deposition

☒ Confirm that both raw and final processed data have been deposited in a public database such as GEO.

☒ Confirm that you have deposited or provided access to graph files (e.g. BED files) for the called peaks.

Data access links<br>*May remain private before publication.*	Datasets generated in this study have been deposited in the Gene Expression Omnibus under accession number GSE163458.
Files in database submission	Files available in the database submission:<br><br>Raw data files<br>HUVEC_YAP_ChIPseq.bw<br>HUVEC_TAZ_ChIPseq.bw<br>HUVEC_TEAD1_ChIPseq.bw<br>HUVEC_pooled_input.bw<br><br>Processed ChIP seq file<br>GSE163458_RAW.tar
Genome browser session<br>(e.g. UCSC)	No longer applicable.

Methodology

Replicates	Pooled chromatin from endogenous YAP, TAZ or TEAD1 pulldown.
Sequencing depth	Illumina sequencing libraries were prepared from the ChIP and Input DNAs by the standard consecutive enzymatic steps of end-polishing, dA-addition, and adaptor ligation.<br>After a final PCR amplification step, the resulting DNA libraries were quantified and sequenced on Illumina's NextSeq 500 (75 nt reads, single end).<br><br>HUVEC-Ctrl_YAP1 33641838 reads<br>HUVEC-Ctrl_TAZ 44410668 reads<br>HUVEC-Ctrl_TEAD1 51197463 reads<br>HUVEC-Pooled Input 38791401 reads
Antibodies	YAP1 (Abcam, ab52771)<br>TAZ (Sigma, HPA007415)<br>TEAD1 (Cell Signaling Technology, #12292BF)
Peak calling parameters	Peak locations were determined using the MACS algorithm (v2.1.0) with a cutoff of p-value = 1e-7. Peaks that were on the ENCODE blacklist of known false ChIP-Seq peaks were removed.
Data quality	Data quality was assessed with the FastQC quality-control tool for high throughput sequence data.
Software	MACS<br>BWA (v0.7.12)<br>bcl2fastq2 (v2.20)<br>Samtools (v0.1.19)<br>BEDtools (v2.25.0)<br>wigToBigWig (v4)
