# Supplementary figures and images for: A YAP/TAZ-TEAD signalling module links endothelial nutrient acquisition to angiogenic growth
Source: Nat Metab. 2022 Jun 20;4(6):672–82. doi: 10.1038/s42255-022-00584-y (PMC9236904; doi:10.1038/s42255-022-00584-y)

Source Data Figure 1

Fig. 1b

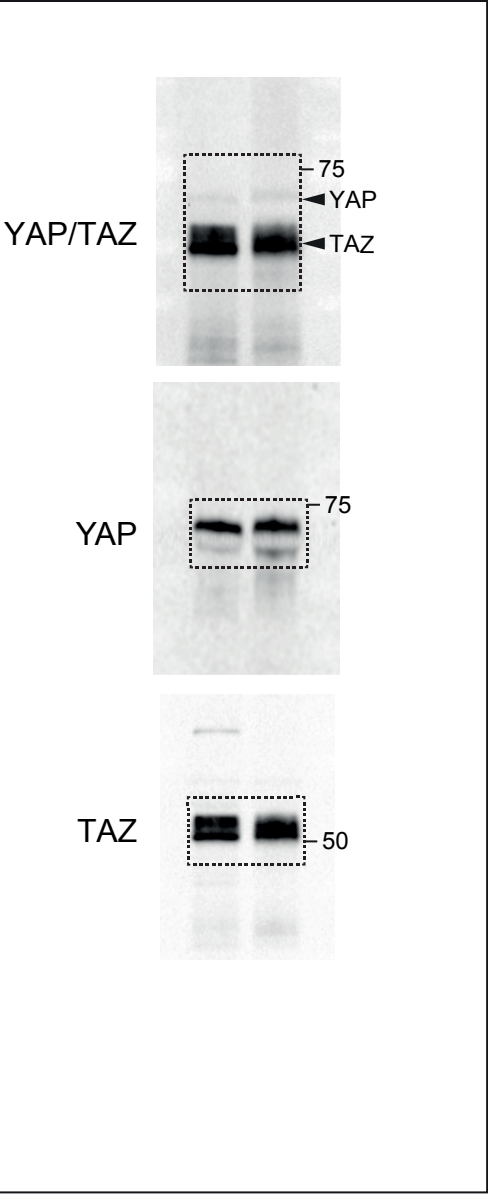

Fig. 1d

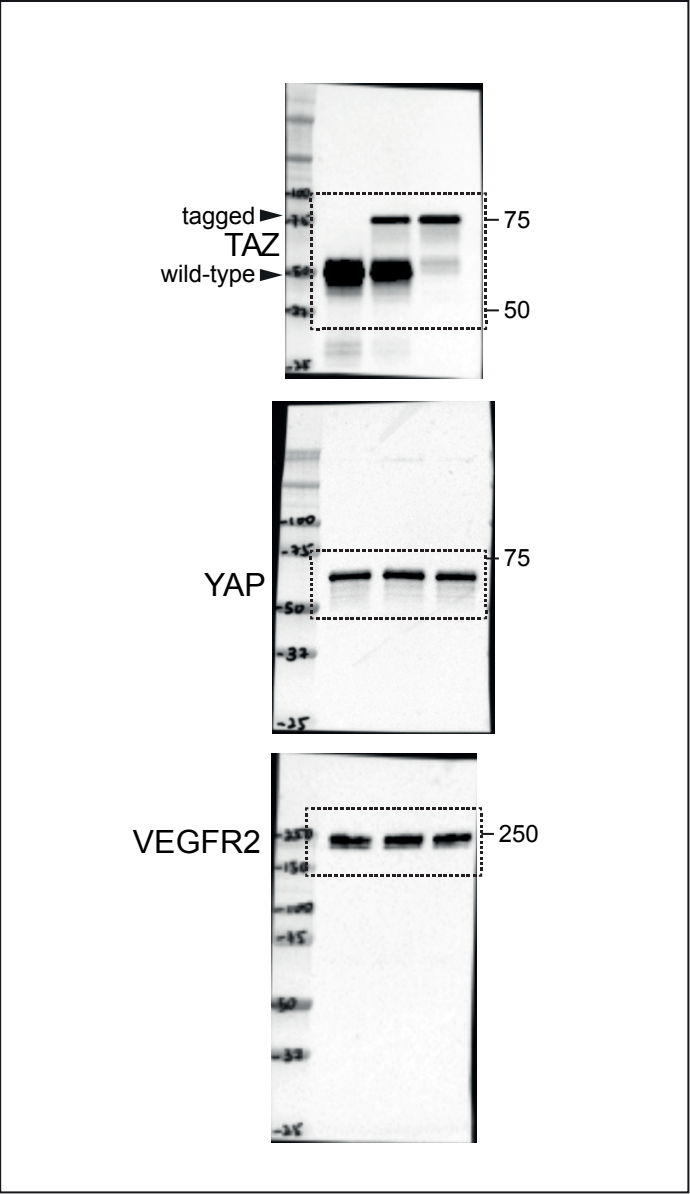

Supplement: Source Data Fig. 1 — Unprocessed blots. [file 42255_2022_584_MOESM10_ESM.pdf]

Source Data Figure 2

Fig. 2c

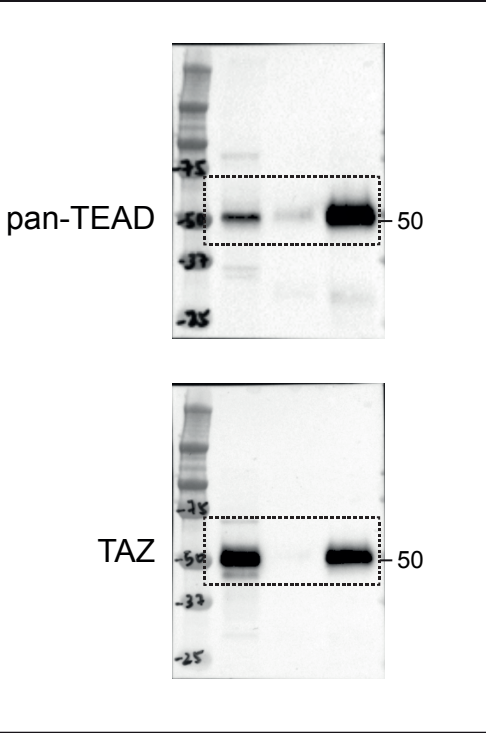

Fig. 2d

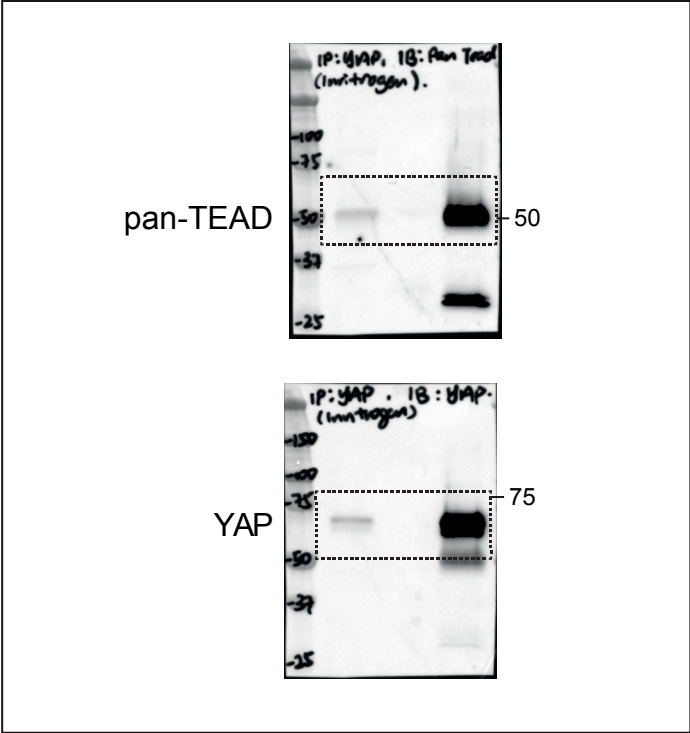

Supplement: Source Data Fig. 2 — Unprocessed blots. [file 42255_2022_584_MOESM12_ESM.pdf]

Source Data Figure 3

Fig. 3f

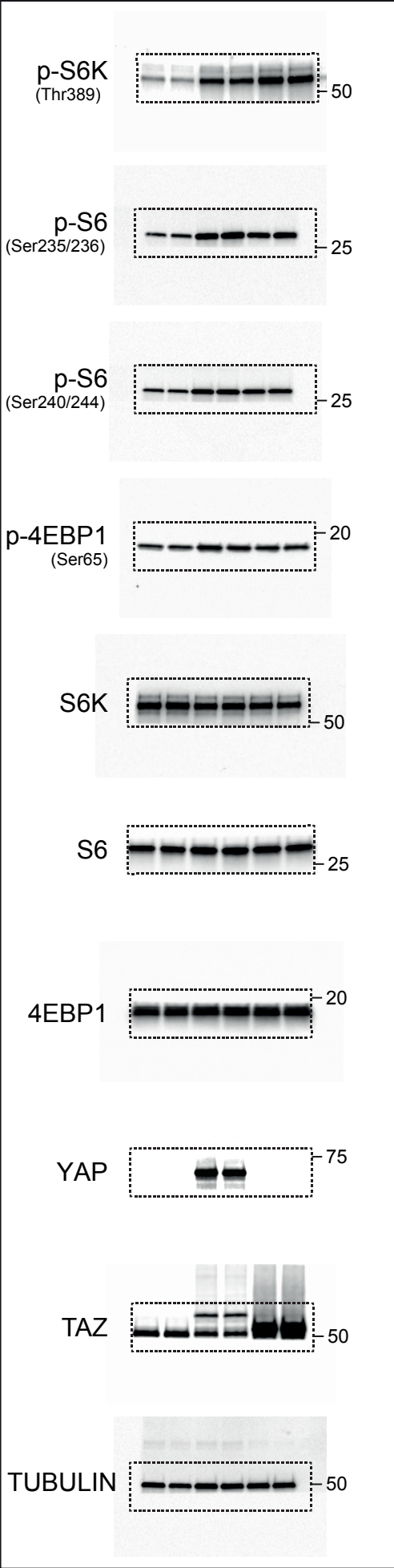

Fig. 3g

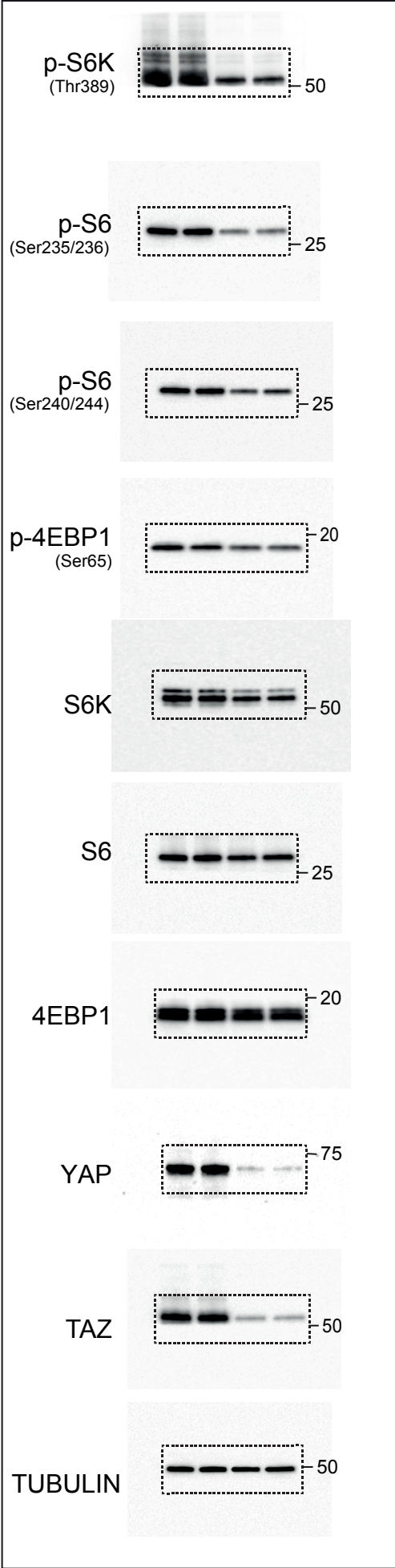

Fig. 3h

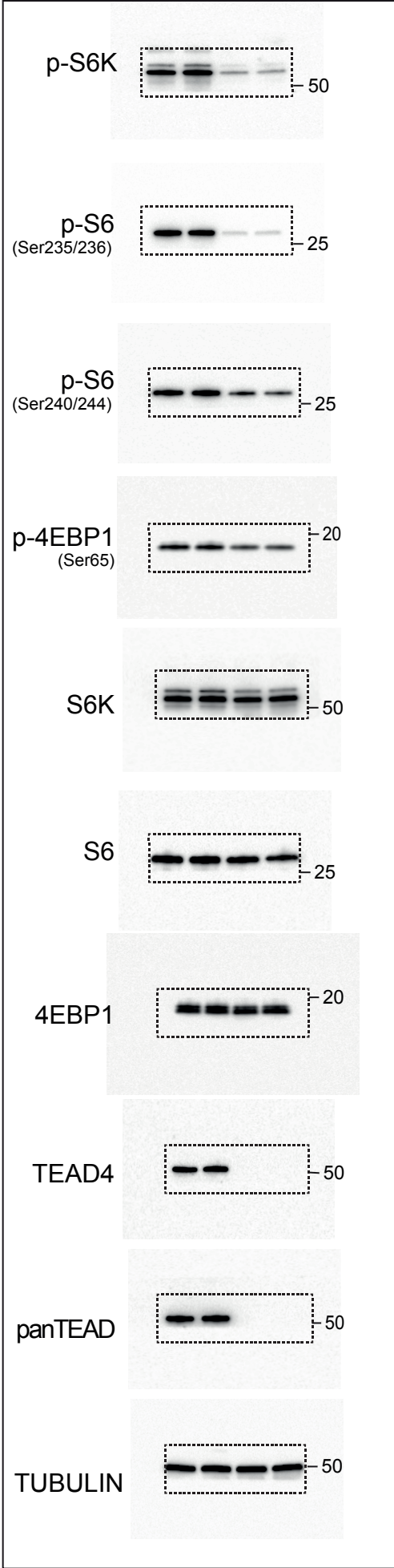

Source Data Figure 3

Fig. 3l

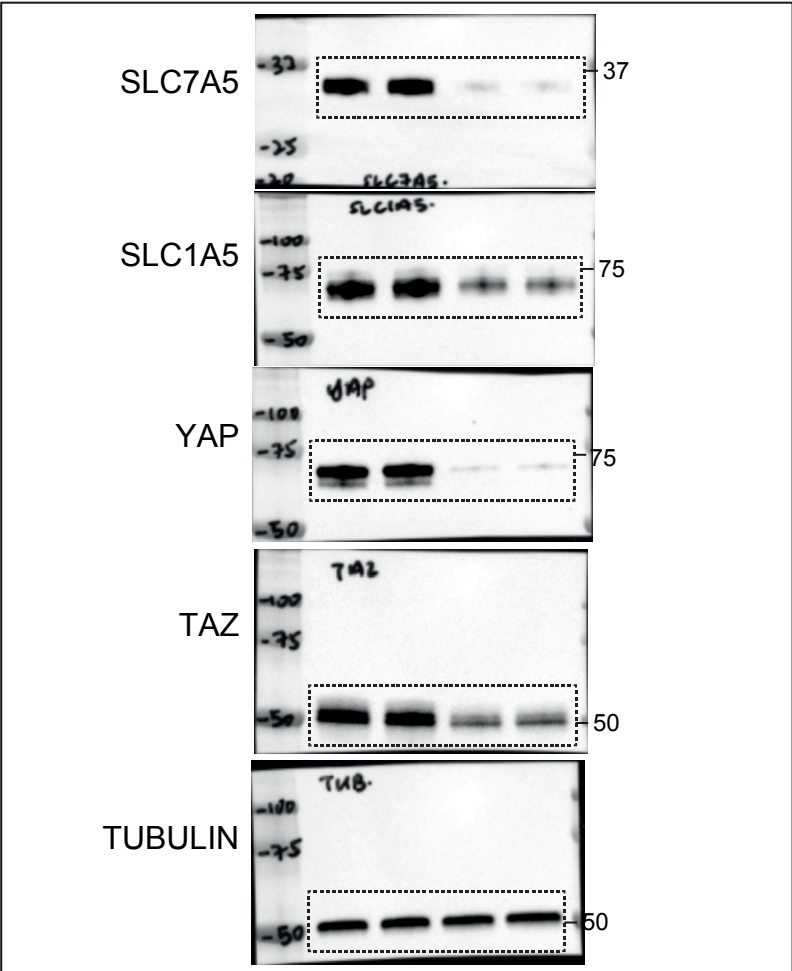

Fig. 3m

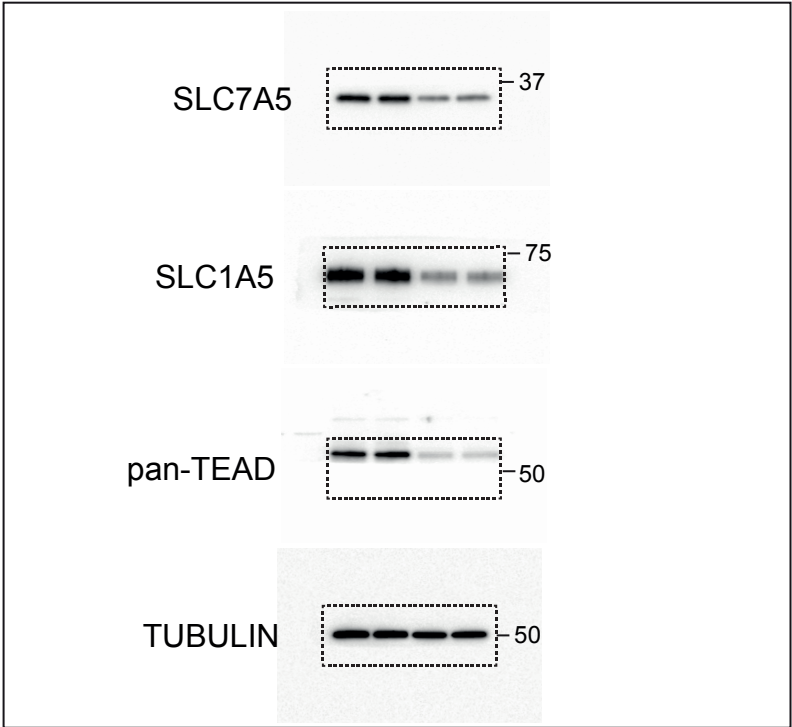

Fig. 3o

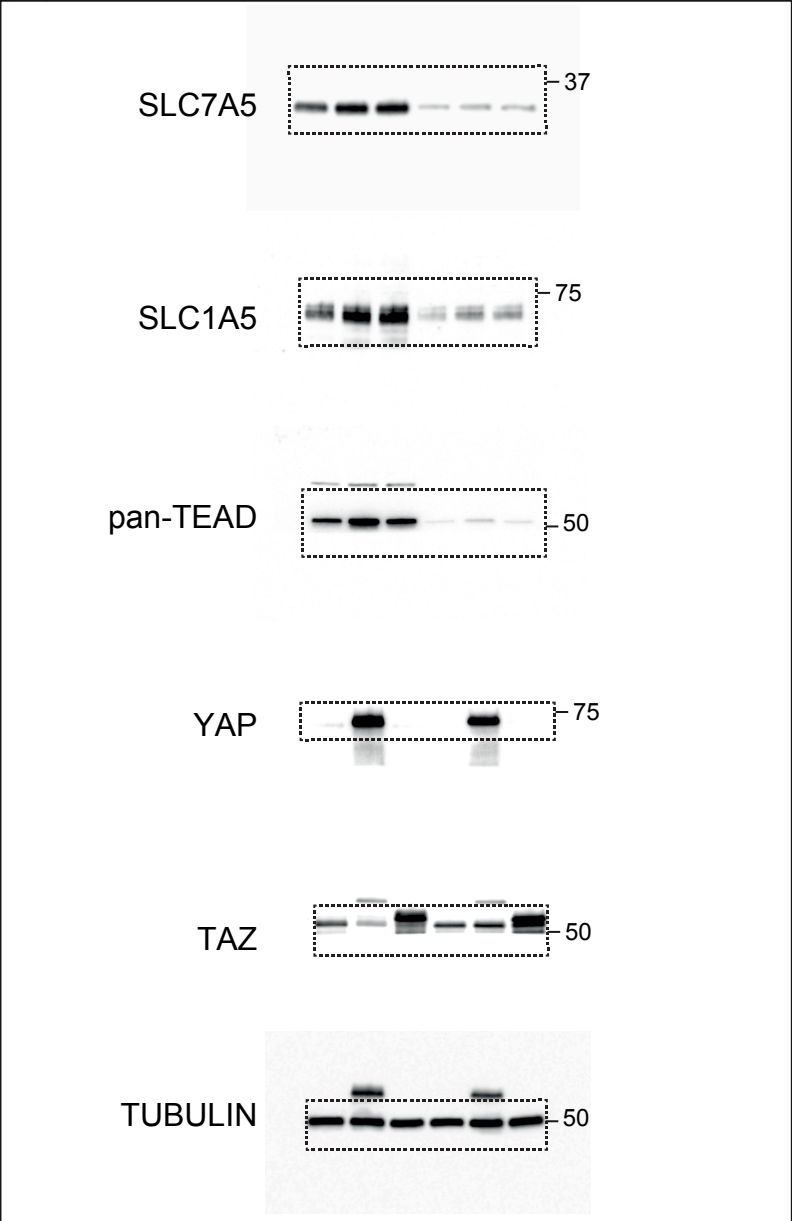

Fig. 3n

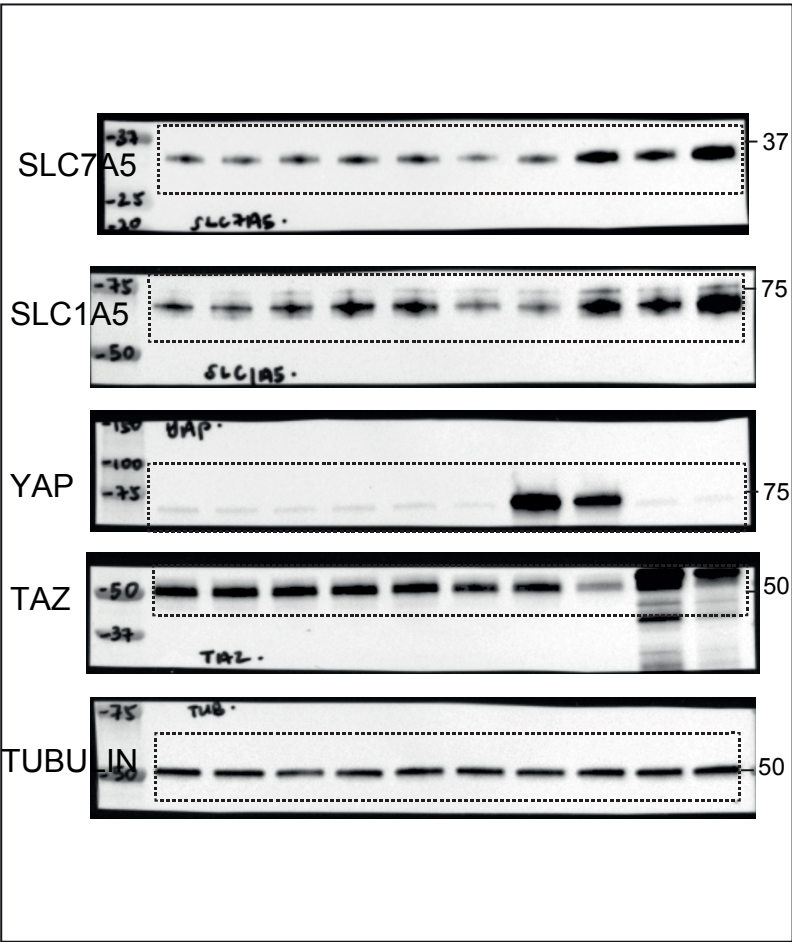

Supplement: Source Data Fig. 3 — Unprocessed blots. [file 42255_2022_584_MOESM14_ESM.pdf]

Source Data Figure 4

Fig. 4b

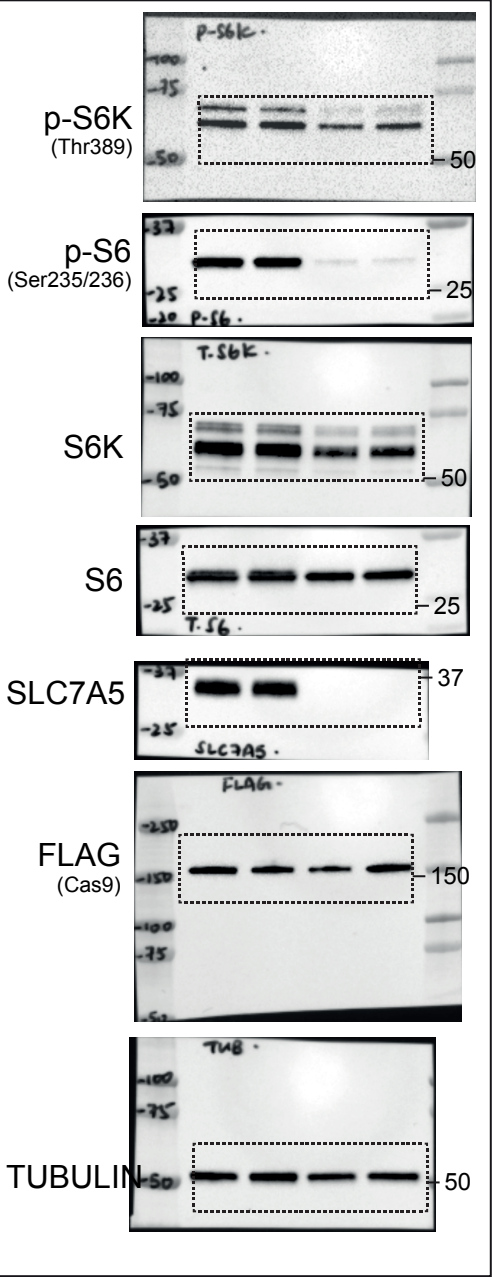

Fig. 4h

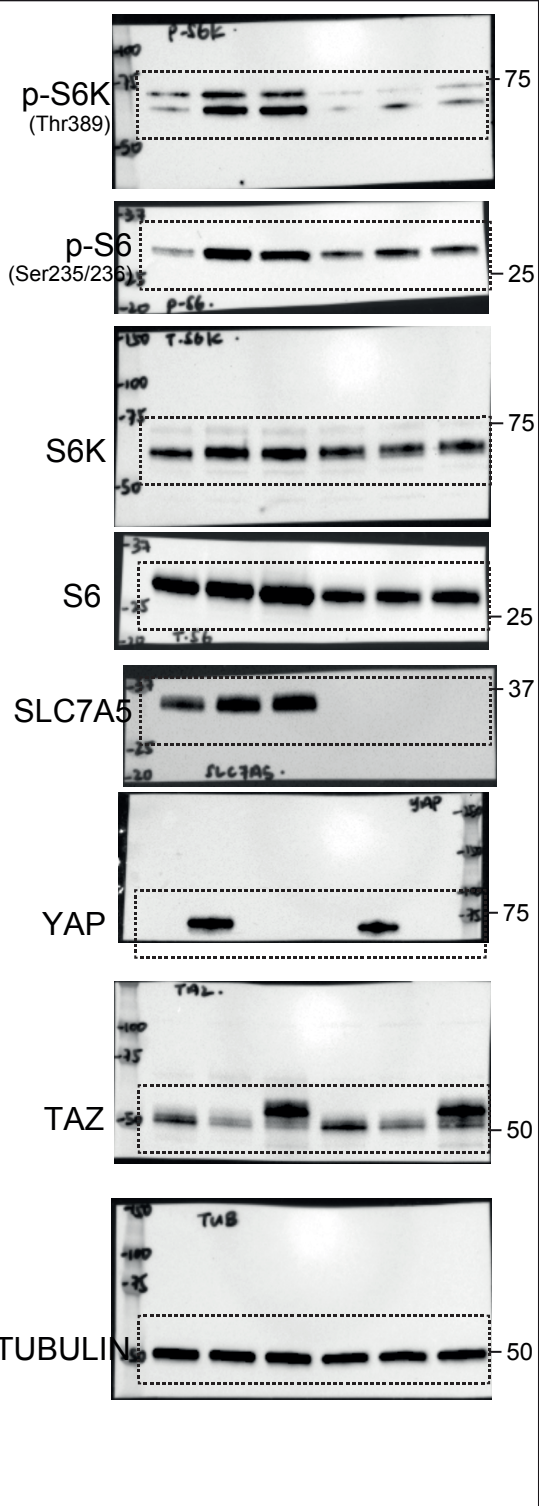

Fig. 4i

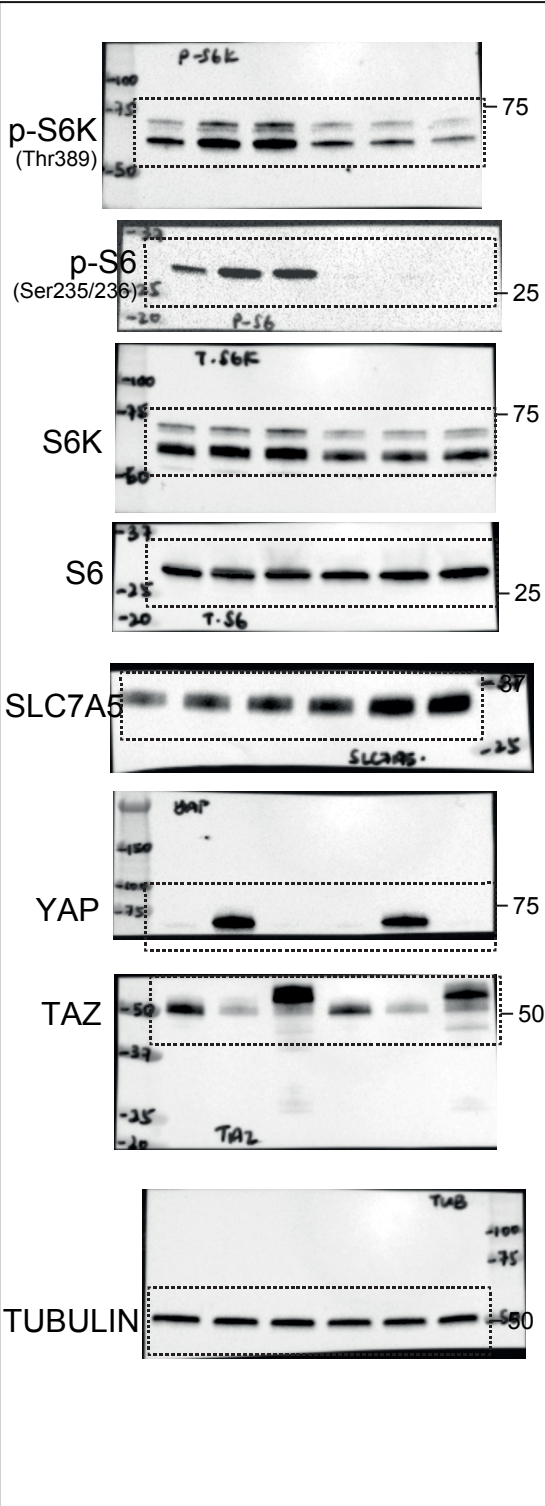

Source Data Figure 4

Fig. 4j

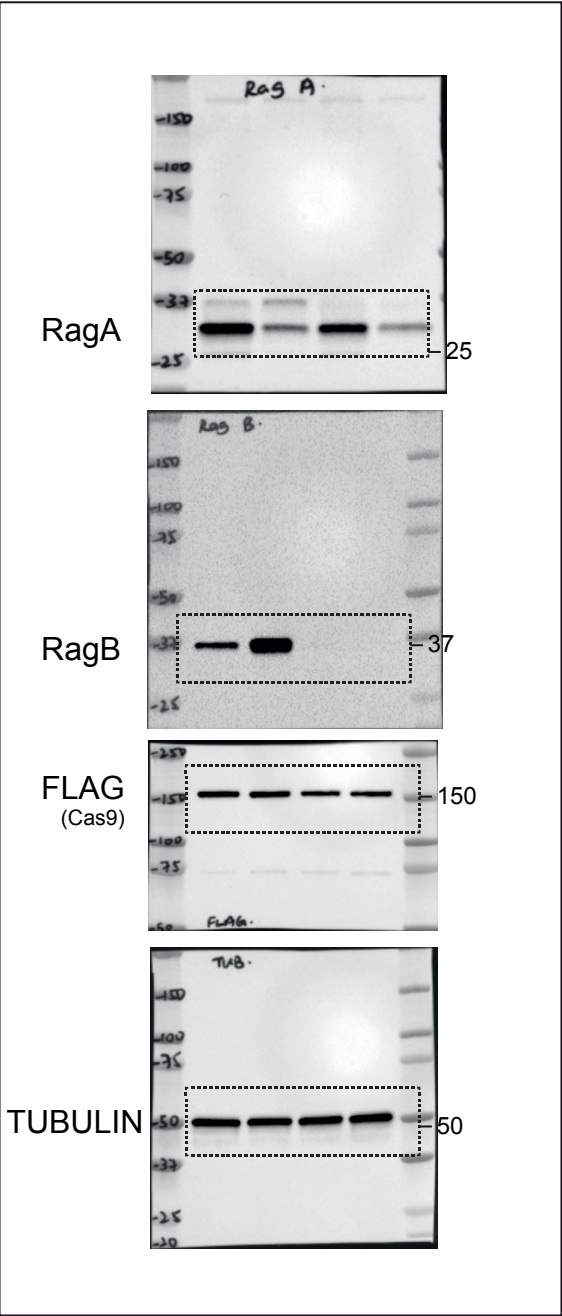

Fig. 4l

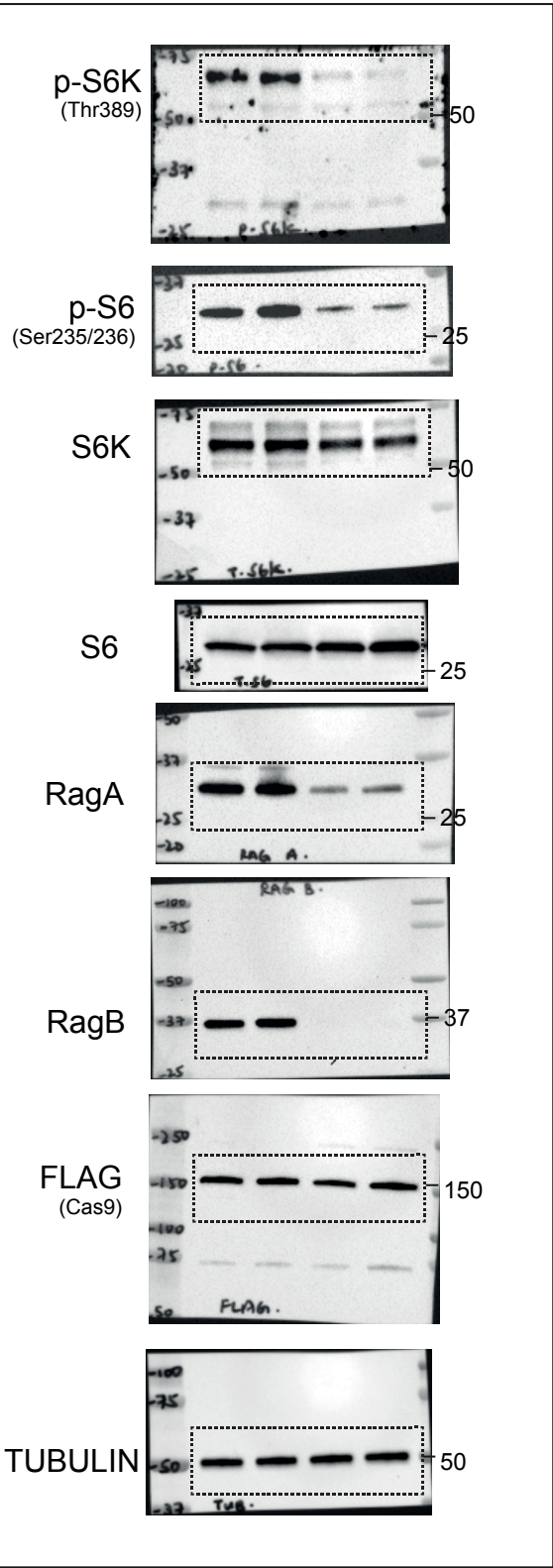

Supplement: Source Data Fig. 4 — Unprocessed blots. [file 42255_2022_584_MOESM15_ESM.pdf]

Source Data Extended Data Figure 2

Figure S2b

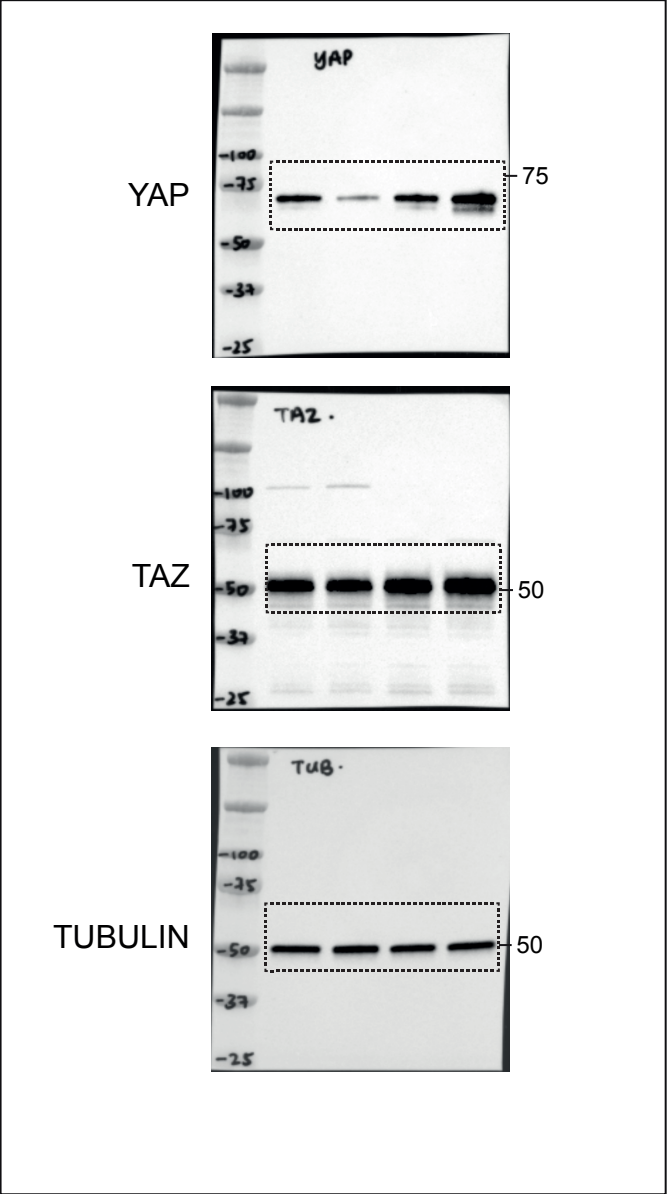

Figure S2d

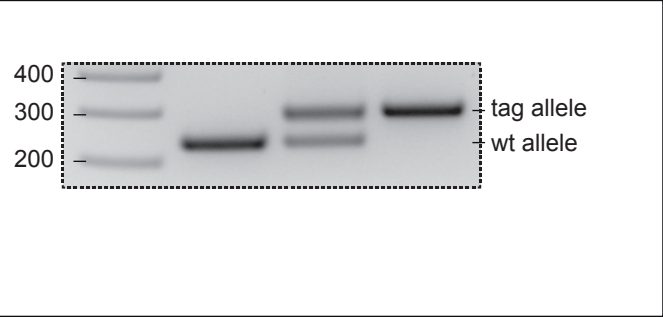

Supplement: Source Data Extended Data Fig. 2 — Unprocessed blots and gels. [file 42255_2022_584_MOESM18_ESM.pdf]

Source Data Extended Data Figure 3

Figure S3b

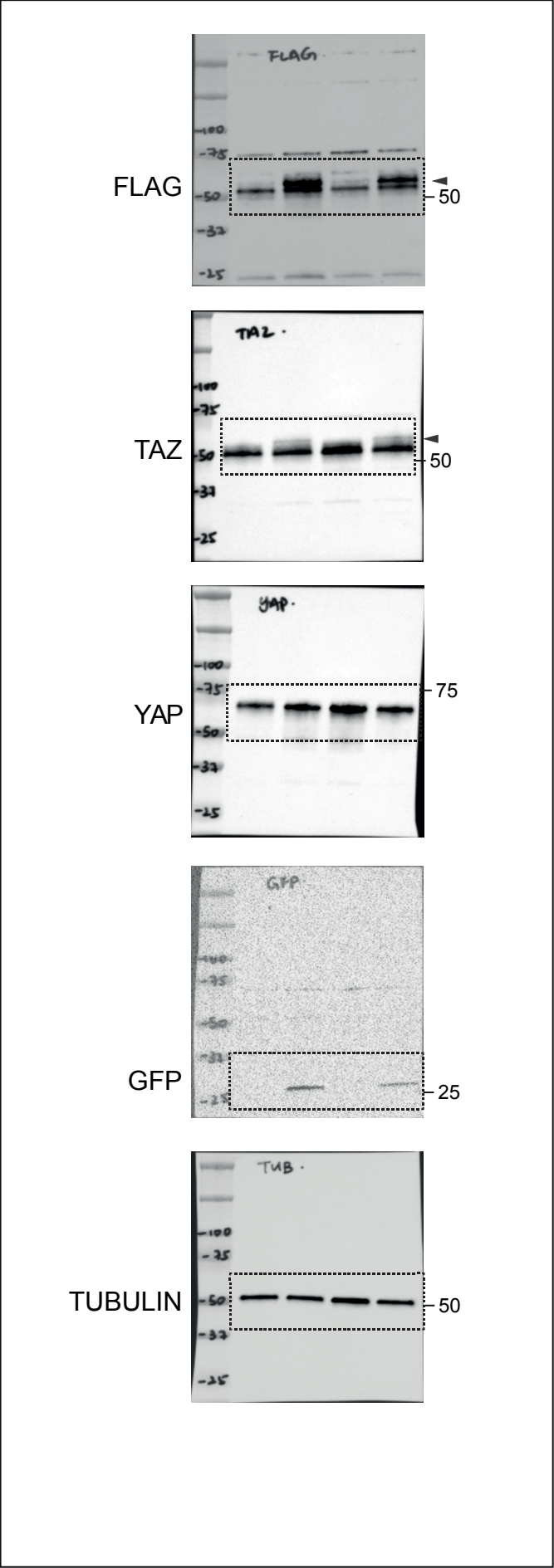

Supplement: Source Data Extended Data Fig. 3 — Unprocessed blots. [file 42255_2022_584_MOESM20_ESM.pdf]

Figure S5a

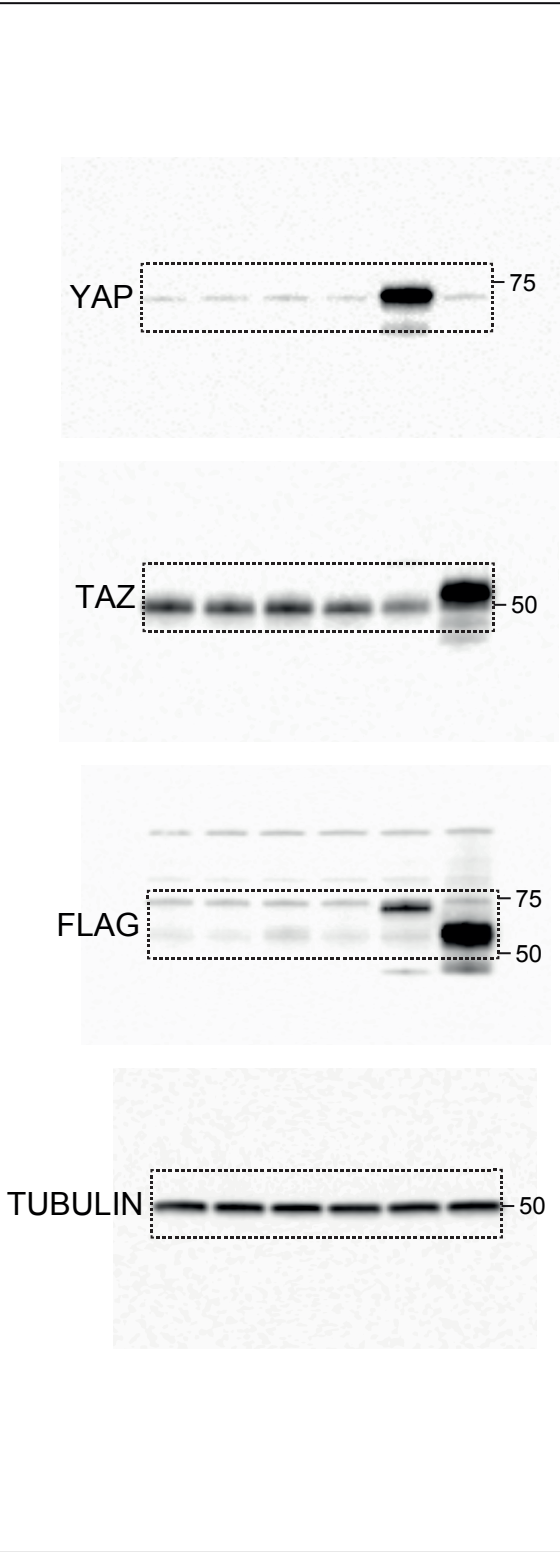

Figure S5e

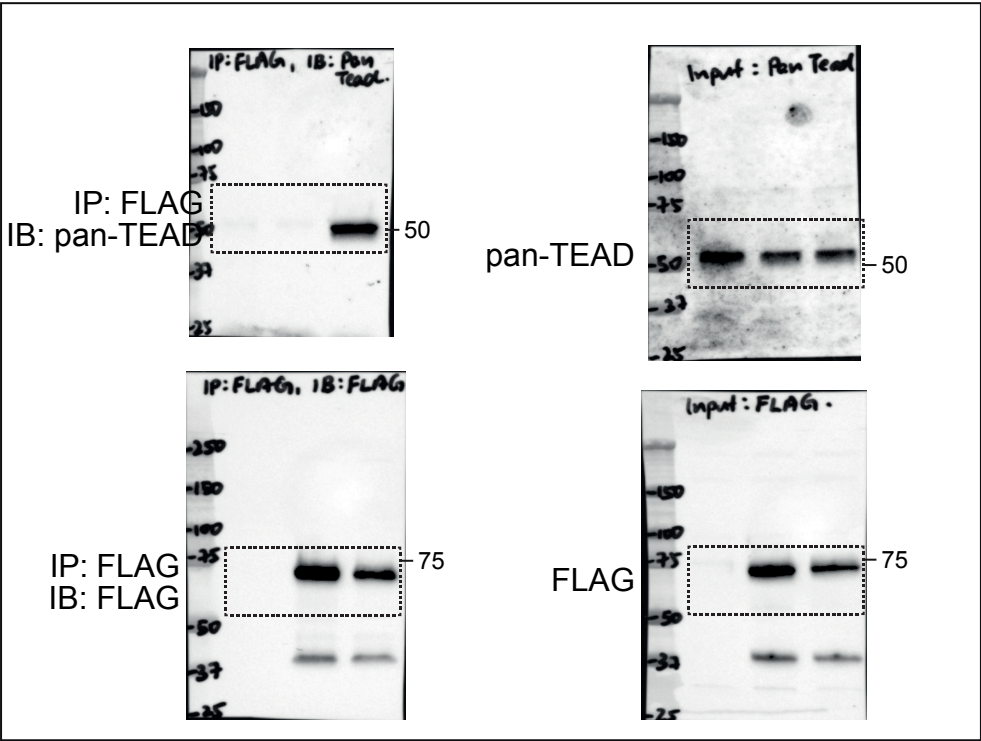

Figure S5f

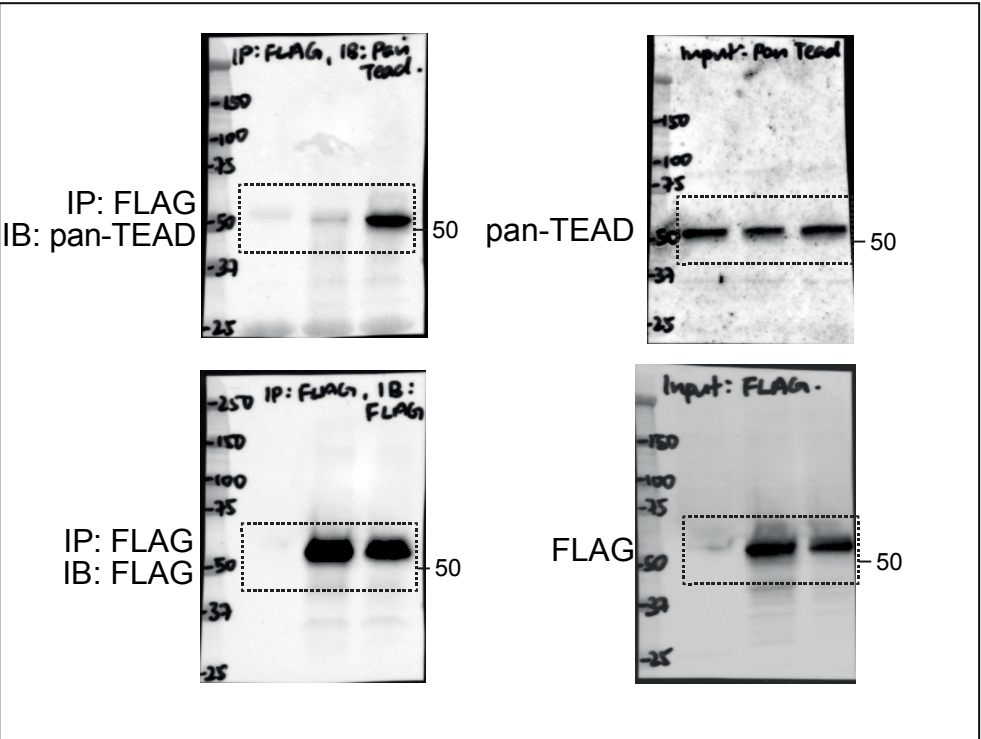

Supplement: Source Data Extended Data Fig. 5 — Unprocessed blots. [file 42255_2022_584_MOESM23_ESM.pdf]

Figure S8b

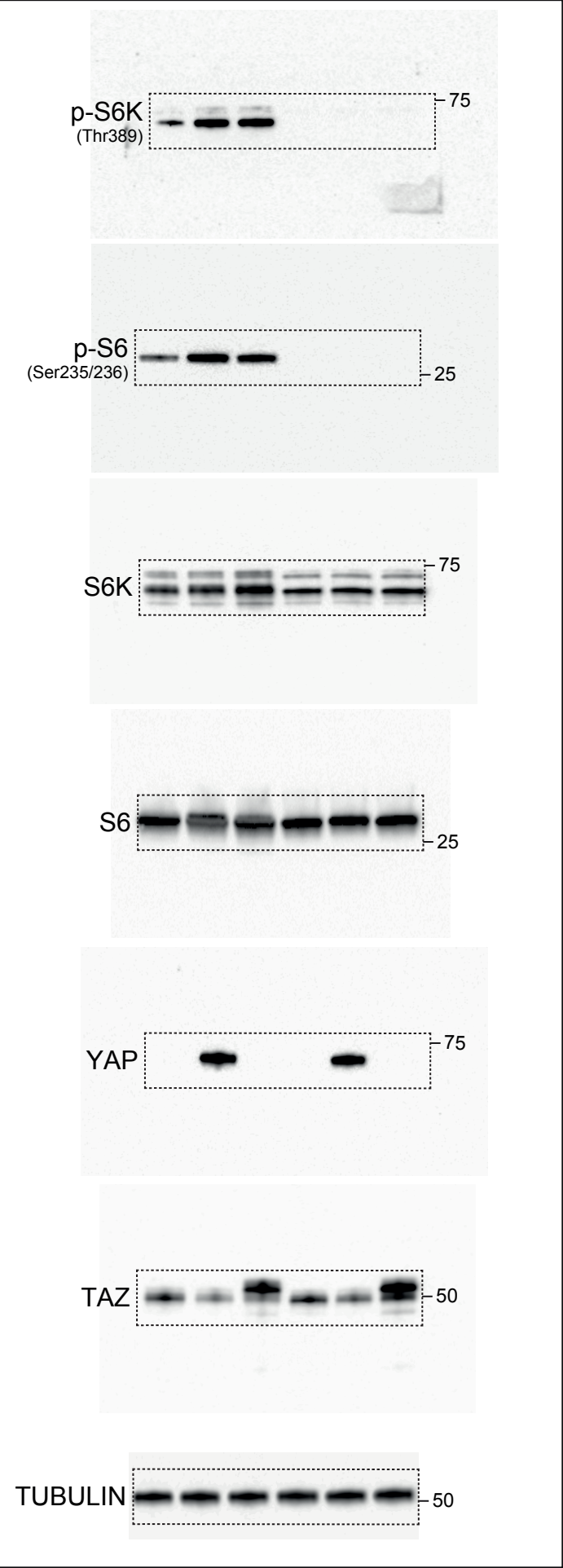

Supplement: Source Data Extended Data Fig. 8 — Unprocessed blots. [file 42255_2022_584_MOESM27_ESM.pdf]

Source Data Extended Data Figure 9

Figure S9i

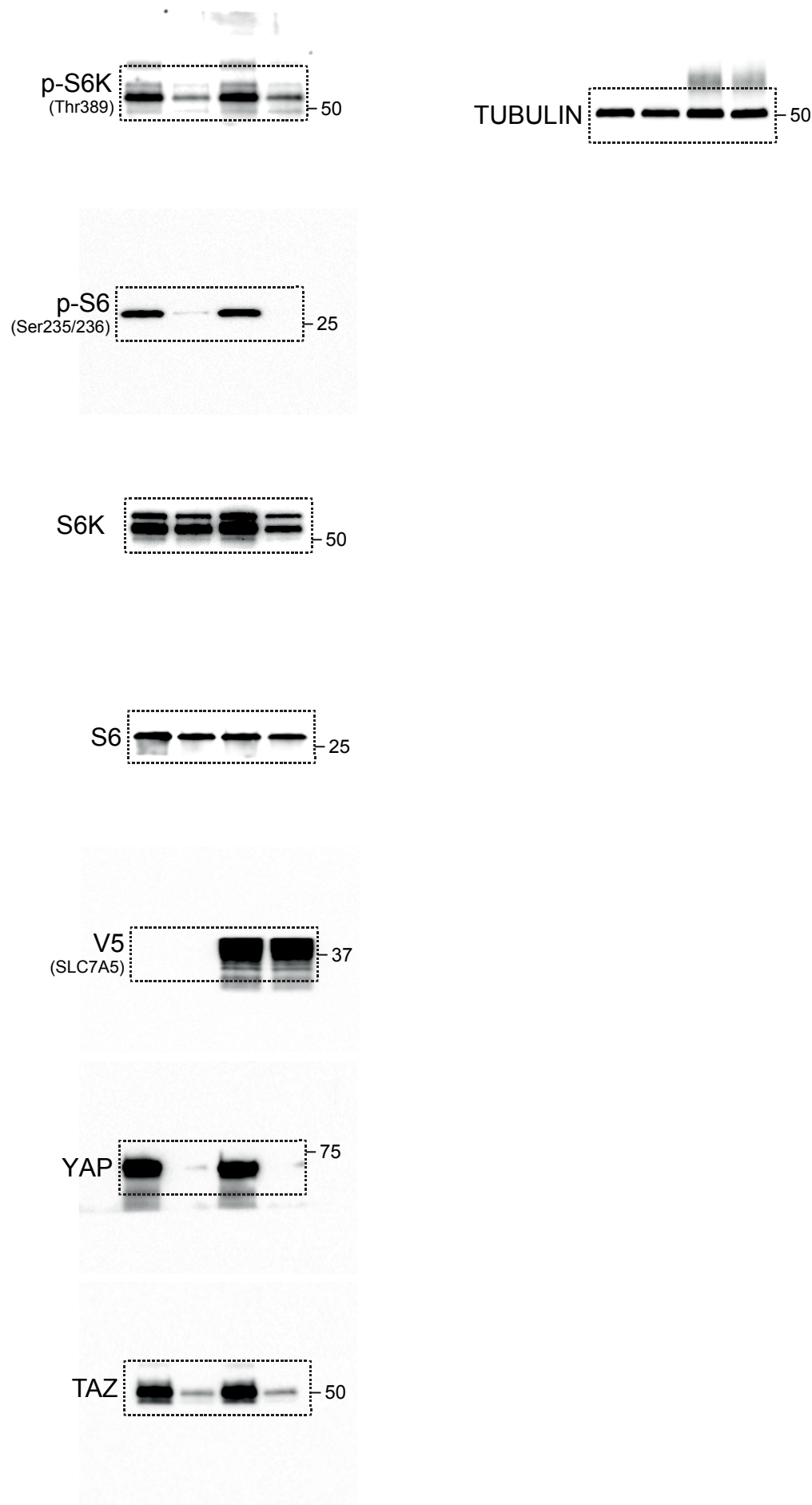

Supplement: Source Data Extended Data Fig. 9 — Unprocessed blots. [file 42255_2022_584_MOESM29_ESM.pdf]

Figure S10b

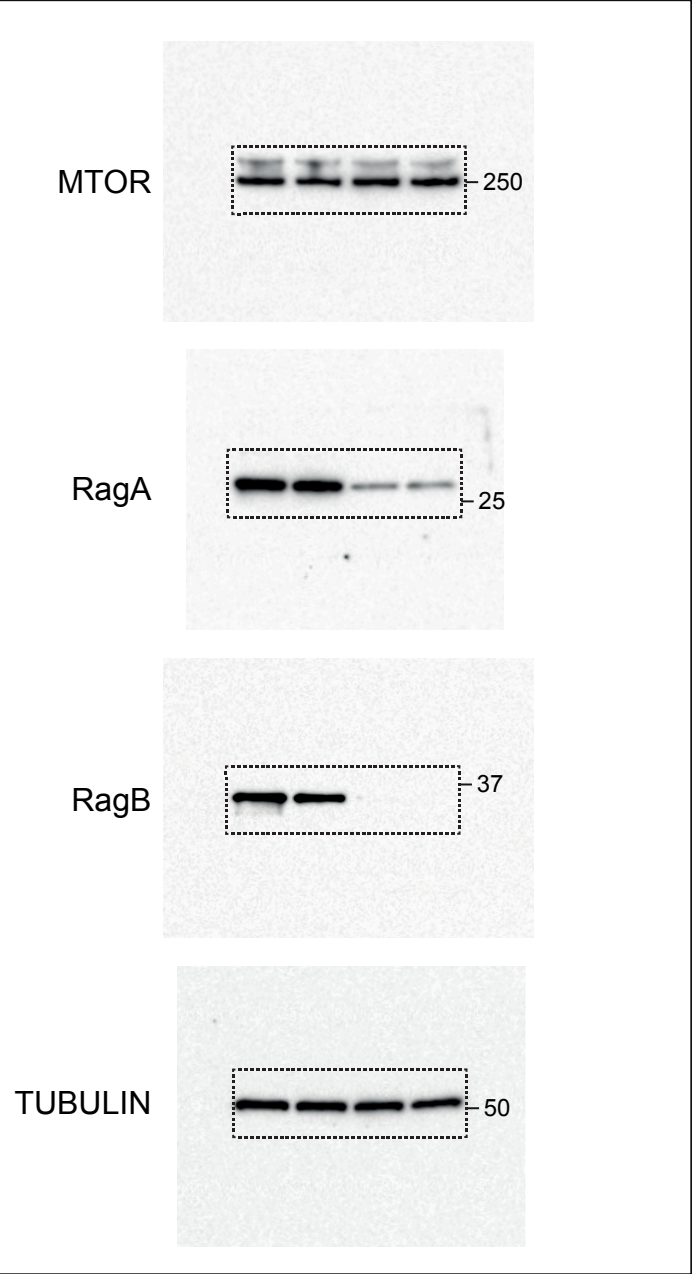

Supplement: Source Data Extended Data Fig. 10 — Unprocessed blots. [file 42255_2022_584_MOESM31_ESM.pdf]
